# Fast and global reorganization of the chloroplast protein biogenesis network during heat acclimation

**DOI:** 10.1093/plcell/koab317

**Published:** 2021-12-27

**Authors:** Raphael Trösch, Fabian Ries, Lisa Désirée Westrich, Yang Gao, Claudia Herkt, Julia Hoppstädter, Johannes Heck-Roth, Matthieu Mustas, David Scheuring, Yves Choquet, Markus Räschle, Reimo Zoschke, Felix Willmund

**Affiliations:** 1 Molecular Genetics of Eukaryotes, University of Kaiserslautern, Kaiserslautern 67663, Germany; 2 Max Planck Institute of Molecular Plant Physiology, Potsdam-Golm 14476, Germany; 3 Biologie du Chloroplaste et Perception de la Lumieère Chez les Microalgues, Institut de Biologie Physico-Chimique, UMR CNRS/UPMC, Paris 7141, France; 4 Plant Pathology, University of Kaiserslautern, Kaiserslautern 67663, Germany; 5 Molecular Genetics, University of Kaiserslautern, Kaiserslautern 67663, Germany

## Abstract

Photosynthesis is a central determinant of plant biomass production, but its homeostasis is increasingly challenged by heat. Little is known about the sensitive regulatory principles involved in heat acclimation that underly the biogenesis and repair of chloroplast-encoded core subunits of photosynthetic complexes. Employing time-resolved ribosome and transcript profiling together with selective ribosome proteomics, we systematically deciphered these processes in chloroplasts of *Chlamydomonas reinhardtii.* We revealed protein biosynthesis and altered translation elongation as central processes for heat acclimation and showed that these principles are conserved between the alga and the flowering plant *Nicotiana tabacum*. Short-term heat exposure resulted in specific translational repression of chlorophyll *a*-containing core antenna proteins of photosystems I and II. Furthermore, translocation of ribosome nascent chain complexes to thylakoid membranes was affected, as reflected by the increased accumulation of stromal cpSRP54-bound ribosomes. The successful recovery of synthesizing these proteins under prolonged acclimation of nonlethal heat conditions was associated with specific changes of the co-translational protein interaction network, including increased ribosome association of chlorophyll biogenesis enzymes and acclimation factors responsible for complex assembly. We hypothesize that co-translational cofactor binding and targeting might be bottlenecks under heat but become optimized upon heat acclimation to sustain correct co-translational protein complex assembly.

## Teaser

Heat exposure at 40°C leads to specific temporary changes of chloroplast translation, majorly affecting antenna proteins of the photosynthesis machinery.

##  

**Figure:**
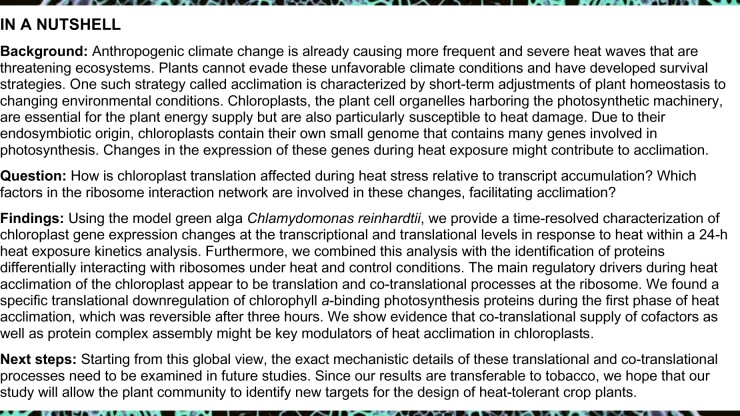


## Introduction

Global warming is a major threat to our food supply caused by intensifying heat waves. An increase of 4°C in average temperatures may lead to an 87% risk of over 10% loss of the global maize (*Zea mays*) harvest (Tigchelaar et al., 2018). Yield is proportional to the level of photosynthesis, and a detailed understanding of photosynthetic acclimation to heat exposure is required for the targeted design of more stress-tolerant crops (Hu et al., 2020; Kleine et al., 2020). Heat exposure leads to various cellular changes such as altered protein homeostasis, cell-cycle arrest, increased membrane fluidity, the switch from regular to stress metabolism, and modified photosynthetic performance (Schroda et al., 2015; Le Breton and Mayer, 2016). The primary effects of heat on chloroplasts and hence photosynthesis are protein denaturation and aggregation, as well as increased membrane fluidity, the former being counteracted by increased accumulation of molecular chaperones and the latter by higher biosynthesis of saturated fatty acids such as digalactosyldiacylglycerol (Hong and Vierling, 2000; Queitsch et al., 2002; Schroda et al., 2015; Rütgers et al., 2017). From work on bacterial and cytosolic translation, heat is known to interfere with protein biosynthesis on many levels, from the stability and folding of RNA structures to altered kinetics of translation initiation and local pausing of elongation (Shalgi et al., 2013; Lukoszek et al., 2016; Zhang et al., 2017). Both cytosolic and chloroplast translation are necessary for thermotolerance (Tanaka et al., 2000). Indeed, the accumulation of the chloroplast translation elongation factor Tu (EF-Tu) is induced by heat stress and is required for thermotolerance in maize (Bhadula et al., 2001). Moreover, knock-down mutants of *EF-Tu* are highly sensitive to heat and show reduced chloroplast translation (Li et al., 2018). However, the effects of heat on the biosynthesis of individual plastid proteins are barely understood. This line of inquiry may be of high relevance for heat acclimation of photosynthesis, since several essential core subunits of the major photosynthetic complexes, photosystem I and II (PSI and PSII), the cytochrome *b*_6_*f* complex, ATP synthase and ribulose-1,5-bisphosphate-carboxylase/-oxygenase (RuBisCO), are locally produced in plastids. Regulation of protein biosynthesis is a key step during plastid gene expression (Eberhard et al., 2002; Zoschke and Bock, 2018), and increased protein biosynthesis is important for the fast repair of the photosynthesis machinery during acclimation to high light (Schuster et al., 2019; Chotewutmontri and Barkan, 2020). One of the major complexes performing the light reaction, PSII, was suggested to be heat-sensitive (De Ronde et al., 2004); in fact, the PSII core subunit PsbA (D1 protein) is cleaved into two fragments by the FtsH protease even under moderate heat stress (Yoshioka et al., 2006). However, other studies have reported that PSII is only heat-sensitive under nonphysiological temperatures (Sharkey et al., 2005; Sharkey and Zhang, 2010) and heat damage to the photosystems under nonlethal conditions is still debated (Schroda et al., 2015). However, chlorophyll loss due to heat exposure is well documented (Allakhverdiev et al., 2008). In *Synechocystis* sp. PCC 6803, most chlorophyll *a* molecules bound to reaction center subunits are de-esterified during repair of PSII (Vavilin and Vermaas, 2007). Chlorophyll de-esterification and re-esterification are necessary for chlorophyll *a* salvage during the repair process (Vavilin and Vermaas, 2007). Recently, a chlorophyll dephytylase 1 (CLD1) from Arabidopsis (*Arabidopsis thaliana*) was shown to be required for thermotolerance (Lin et al., 2016). Reduced expression of *CLD1* led to a reduction in thermotolerance, and the temperature-sensitive mutant allele *cld1-1* exhibited a bleaching phenotype under high temperature due to chlorophyllide accumulation (Lin et al., 2016). Chlorophyll synthase is essential for the re-esterification of the phytyl chain, with temperature-sensitive mutants showing similar heat-induced bleaching phenotypes (Lin et al., 2014). In sum, heat acclimation of chloroplasts may rely on adjusted plastid protein biosynthesis and chlorophyll reutilization for repair and reorganization of protein complexes involved in photosynthesis during heat exposure, a response that is still not well understood. We thus set out to monitor translational regulation in a time-resolved manner during acclimation to high temperature in Chlamydomonas (*Chlamydomonas reinhardtii*) cells. Chlamydomonas is a well-suited model for observing heat acclimation in plant cells, since its plastidic biochemical processes are conserved with those of land plants, and heat can be homogenously applied to all cells of the culture without simultaneously causing drought stress (Schroda et al., 2015). Chlamydomonas cultures were exposed to non-lethal 40°C, and chloroplast transcript accumulation and translation were assayed by targeted chloroplast ribosome profiling (Trösch et al., 2018). We observed early, intermediate and late changes, suggesting the sequential nature of heat acclimation on the translational level. At time points with detectable acclimation reactions, we identified ribosome-associated factors in parallel to reveal putative drivers of plastid heat acclimation.

## Results

### Subcellular relocation of chloroplast ribosomes during heat exposure

**Figure 1:**
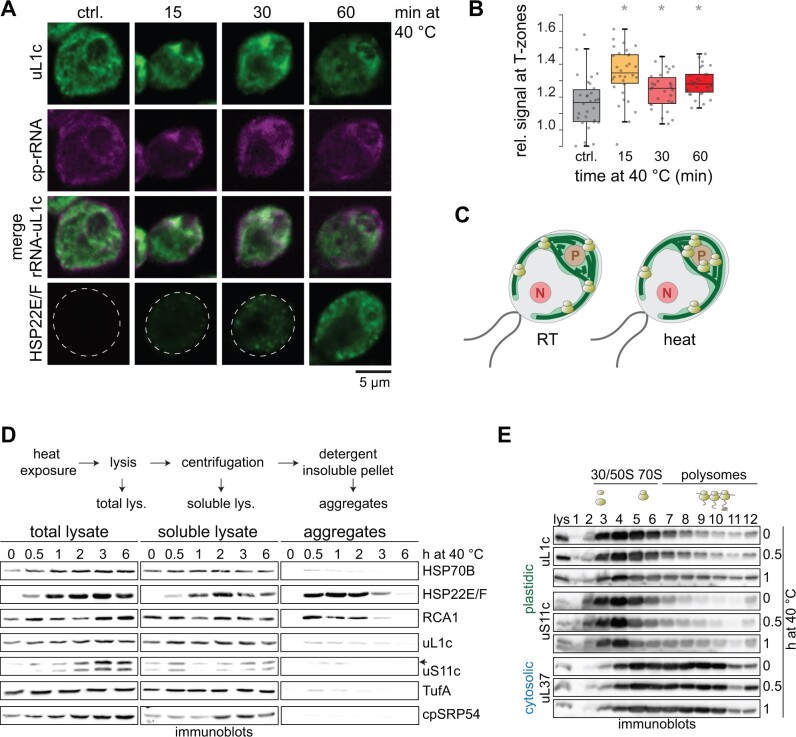
Distribution of chloroplast ribosomes during heat acclimation. A, Representative confocal microscopy images of Chlamydomonas cells during heat exposure at 40°C. Chloroplast ribosomes were visualized by staining with antibodies against uL1c (FITC, green) and a FISH probe against chloroplast rRNA (magenta). Detection of HSP22E/F (FITC, green) serves as control for heat-induced protein aggregates in the chloroplast. Contrast for the magenta channel was slightly but equally adjusted in all panels. Additional images are shown in Supplemental Figure S1. B, Boxplots of quantified uL1c immunofluorescence signals adjacent to the pyrenoid (T-zones), relative to uL1c fluorescence signals in the whole cell (assayed for 30 cells). Asterisks indicate significant differences relative to the control, based on Welch’s unequal variances *t*-test with *P* < 0.05. C, Diagram summarizing the redistribution of plastid ribosomes during heat exposure. P, pyrenoid; N, nucleus; light green, chloroplast; dark green lines, thylakoid membranes. D, Distribution of proteins in soluble or insoluble aggregate fractions during heat exposure. Top panel, experimental setup. Total and soluble lysates were loaded on the basis of equal volume, aggregates are in 20-fold excess. Lower panel: immunoblots of total, soluble, and aggregated proteins for the indicated proteins and time points (*n* = 3). Band for uS11c is indicated by an arrow. E, Polysome distribution during heat exposure. Immunoblots of sucrose gradient fractions of control and heat-treated samples with antibodies against plastid ribosomal proteins uL1c and uS11c and cytosolic ribosomal protein uL37 (RPL37). Expected positions of unassembled ribosomal subunits, monosomes and polysomes in the gradient are indicated by the diagrams above the blots (representative for three biological replicates, see “Methods”). Lys = total lysate.

We applied fluorescence in situ hybridization (FISH) and immunofluorescence to localize chloroplast rRNA and plastid ribosomal protein uL1c (nomenclature according to Ban et al. (2014), classically named RPL1 [ribosomal 50S protein L1]) within Chlamydomonas single cells upon heat exposure to examine the suborganellar localization of translation. After 15 min at 40°C, chloroplast rRNA and uL1c localization changed from a diffuse chloroplast pattern to a higher density around lobe junctions (Figure 1, A–C; Supplemental Figure S1), a known site of thylakoid protein biogenesis in Chlamydomonas (Uniacke and Zerges, 2009). At later time points, this ribosome relocation was less severe, possibly due to heat acclimation responses. To assess whether proteins involved in translation may form heat-induced insoluble protein inclusions, we probed total lysates, soluble proteins, and detergent-resistant aggregates by immunoblotting (Figure 1, D). We detected weak signals for the plastid ribosomal proteins uL1c and uS11c (40S ribosomal protein S11 [RPS11]) and the EF-TufA in aggregates, but observed no visible decrease of soluble ribosomal proteins under these conditions. This observation contrasted with the behavior of typical candidates sequestered in heat-induced aggregates, heat-shock protein 22E/F (HSP22E/F) and RuBisCO activase (RCA1; Feller et al., 1998; Rütgers et al., 2017), suggesting that only a minor fraction of chloroplast ribosomes becomes insoluble during heat treatment (Figure 1, D). Another ribosome-associated factor, the chloroplast signal recognition particle 54 (cpSRP54), appeared completely insensitive to aggregation.

Importantly, polysome assays revealed no pronounced general heat-induced collapse of plastid translation. Instead, polysomal migration of these ribosomes might even slightly increase during heat acclimation, suggesting that translation elongation might be altered in the acclimation phase (Figure 1, E).

### Heat leads to profound changes in translation output of specific chloroplast transcripts

**Figure 2:**
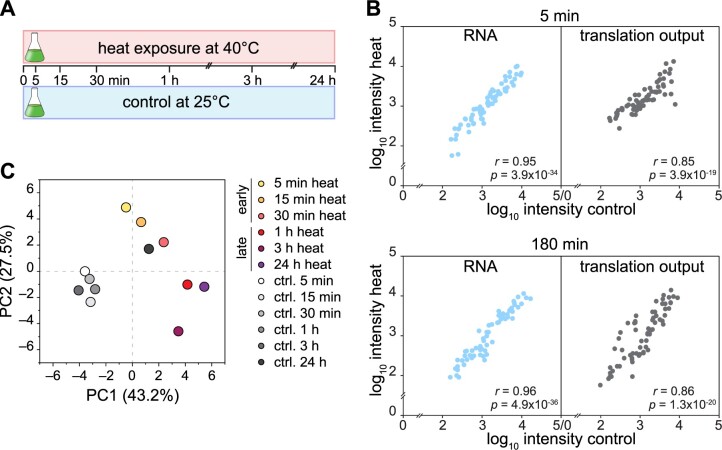
Metagene view of chloroplast mRNA changes and translation profile during heat acclimation. A, Schematic diagram of the experimental setup: Cells were exposed to heat at 40°C and harvested at the indicated time points. Control cultures were treated at room temperature (25°C). B, Scatterplot comparing the translation output and total RNA accumulation between control and 5- or 180-min heat exposure. The average ribosome footprint (translation output) and transcript (RNA) abundances were calculated for each RF and plotted on a Log_10_ scale; *x*- and *y*-axes are broken for better visualization. Pearson’s correlation coefficients and ANOVA’s *P*-values (stating the significance of the regression) are given in the plot. C, PCA of translation output of heat-treated and control samples. A PCA plot representing all individual replicates is shown in Supplemental Figure S3.

Next, we investigated the effects of heat exposure on global chloroplast translation output and transcript accumulation in a time-resolved manner. To this end, we performed ribosome profiling (Trösch et al., 2018) on mixotrophically grown Chlamydomonas cells and collected samples over 24 h of heat exposure (Figure 2, A). Samples at respective time points of heat treatment and control were analyzed in parallel (see “Methods”). For each chloroplast reading frame (RF), we calculated translation output by averaging all ribosome footprint values per RF. RNA accumulation was determined accordingly. We also calculated translation efficiency (TE) as the ratio between translation output and mRNA levels. The data from three independent biological replicates showed high reproducibility (Supplemental Figure S2; Supplemental Data Set S1).

RNA accumulation was highly similar between control and heat-treated cells, as exemplified for the 5- and 180-min time points (correlation coefficients of 0.95 and 0.96, respectively, Figure 2, B). In contrast, translation deviated more strongly for some genes between heat-exposed cells and the control (correlation coefficients of 0.85 [at 5 min] and 0.86 [at 180 min], Figure 2, B). Principal component analysis (PCA) on translation output data showed that the controls cluster together well, while heat-exposed samples cluster in an early (5–30 min) and late (1–24 h) group (Figure 2, C; Supplemental Figure S3). Note that the last time point of the control is shifted in the PCA and shows lower correlations to the other control time points, possibly since the culture might have reached stationary phase after 24 h of growth (Figure 2, C; Supplemental Figures S2, S3).

**Figure 3:**
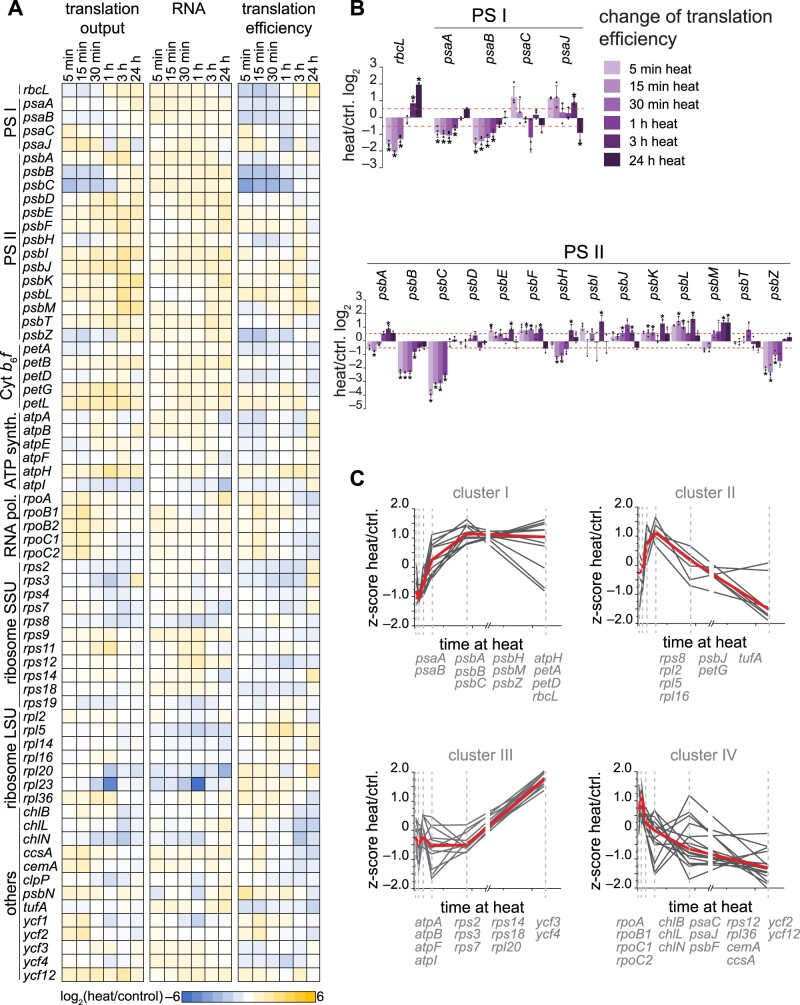
Gene-specific changes of chloroplast mRNA accumulation and translation output during heat acclimation. A, Heatmap represents the relative average values of translation output, transcript accumulation (RNA) and TE; each row represents one gene, as indicated next to the heatmap. All values are plotted on a Log_2_ scale and are colored as indicated by the color scale. B, Mean ratios of TE changes (heat over control per time point of the kinetics) on a Log_2_ scale for *rbcL*, PSI, and PSII subunits. Data for all chloroplast genes are given in Supplemental Figure S6. Data are shown as means ± standard deviation. Individual data points are shown as black circles. C, Translation efficiencies (heat/control) over the time course were subjected to ANOVA testing, significantly changing TEs were *Z*-scored-normalized and clustered into four groups (I–IV). The red line represents the median trend for each cluster. Genes for each cluster are given below the graph. Transcripts with nonsignificantly altered TEs are not shown. All plots represent the means of three biological independent replicates (see “Methods”).

We compared translation output, RNA accumulation and TE for each chloroplast RF between heat treatment and the respective control and illustrated the results as a heatmap (Figure 3, A) or column graph (Supplemental Figures S4–S6). Here, we determined relative abundance by normalizing the value of each RF against the average value of all RFs at a given time point and condition. This calculation thus removed global effects of increased temperature on transcription, translation initiation, and elongation, allowing for the analysis of transcript-specific changes during heat acclimation. Indeed, we detected effects for the translation of specific chloroplast transcripts. The strongest decrease in ribosome footprints occurred for the biosynthesis of the two PSII antenna subunits PsbB/CP47 and PsbC/CP43 after only 5 min of heat treatment, with a subsequent recovery of translation after 60 min (Figure 3, A). We also observed lower ribosome footprints for PsbH and PsbZ, two components associated with PsbB/CP47 and PsbC/CP43 in the PSII complex, in the first hour of heat treatment (Figure 3, A), suggesting that PSII complex assembly might influence the translational status.

Altered translation relative to mRNA levels (TE) during heat acclimation was most profound for chloroplast-encoded subunits of the core photosynthesis complexes, with similar downregulation of the inner antenna components of PSI (i.e. PsaA and PsaB) and PSII (i.e. PsbB/CP47 and PsbC/CP43; Figure 3, B). To identify similar heat acclimation patterns for genes with significantly altered TE, we performed an analysis of variance (ANOVA) test over the time series. Unsupervised hierarchical clustering of *Z*-score-normalized TE identified four larger clusters (I–IV) of translational changes over the heat treatment (Figure 3, C). Indeed, functional groups tended to fall into separate clusters, such as proteins of the photosynthesis machinery (Clusters I and II), ATP synthase subunits and several ribosomal proteins (Cluster III) and a more diverse cluster containing RNA polymerase and light-independent protochlorophyllide oxidoreductase (POR) subunits (Cluster IV).

### Altered *psbB* and *psbC* translation elongation and consequences for protein levels

**Figure 4:**
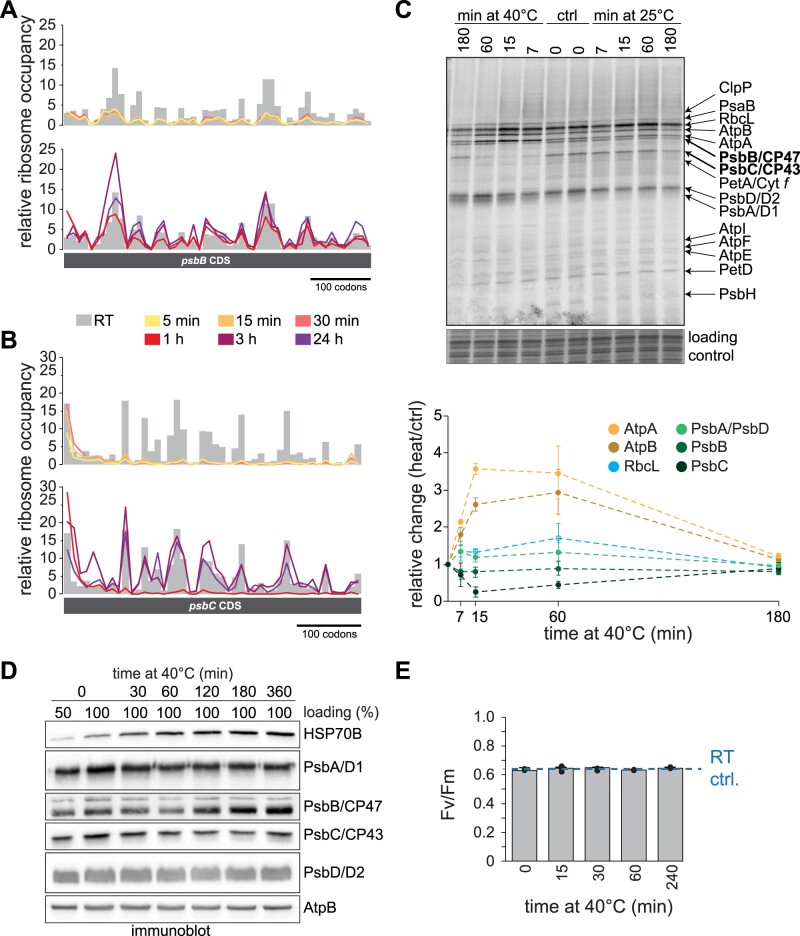
Heat-induced ribosome redistribution on transcripts that encode PSII antenna subunits. A and B, Relative ribosome footprint occupancy, as determined by normalizing single probe values relative to the mean intensity of all plastid ribosome footprints and plotted against the genomic position of the probe on the CDS. Values for control samples are represented as gray columns, values for heat-treated samples are shown as lines. Plots of separate replicates are shown in Supplemental Figure S7. C, Upper panel: Autoradiogram of [^14^C]-acetate pulse labeling of newly translated plastid-encoded proteins at room temperature (25°C) and heat conditions. Cells were transferred to untreated or preheated medium and labeled at the indicated time points for 7 min in the presence of the cytosolic translation inhibitor cycloheximide. Protein bands are assigned according to similar analyses with mutant strains (de Vitry et al., 1989; Lemaire and Wollman, 1989; Girard-Bascou et al., 1992; Minai et al., 2006). Coomassie staining of the same gel serves as loading control. Lower panel: quantification of intensity changes of the indicated nascent polypeptides, relative to the untreated control. Data are shown as means from three experiments with individually grown cells, ±standard deviation. D, Immunoblotting of samples from control and heat-treated Chlamydomonas cells for selected PSII subunits; HSP70B serves as positive control for heat-induction, AtpB serves as loading control. The immunoblot is representative of three replicates from independently grown and processed samples. E, Photosynthetic activity of PSII of Chlamydomonas, as determined by maximum quantum yield of fluorescence (*Fv/Fm*) over the course of a heat kinetics at 40°C. Dashed line indicates the *Fv/Fm* value of control cells, maintained at room temperature (RT ctrl) for the same time. Mean values of three independently grown replicates are shown ±standard deviation.

To reveal putative local changes of ribosome occupancy, possibly causing the reduction of *psbB* and *psbC* translation, we plotted ribosome footprint intensities of the respective probes normalized to the average of all footprints within the RF (Figure 4, A and B). Surprisingly, the reduced translation of *psbB* and *psbC* appeared to be caused by different mechanisms. During early heat exposure, translation of *psbB* showed a homogeneous decrease in ribosome occupancy along the RF, which already partially recovered after 1 h of heat exposure, suggesting a temporal reduction in initiation (Figure 4, A; Supplemental Figure S7, A). For *psbC*, ribosome occupancy was not affected over the first 6–7 probes (covering the first 60 codons) but displayed a very strong reduction downstream for the first hour of heat exposure. It is thus possible that the reduced translation might be caused by elongation arrest once the nascent polypeptide starts to emerge from the ribosomal tunnel exit (Figure 4, B; Supplemental Figure S7, B). [^14^C]-acetate pulse labeling of chloroplast-encoded proteins showed that several nascent polypeptides of abundant proteins (e.g. RbcL, AtpA, and AtpB) increasingly accumulate during heat exposure, which may be the consequence of a general increase in acetate uptake or increased translation rates (Figure 4, C). Translation of the inner core proteins of PSII, PsbA/D1, and PsbD/D2, increased only mildly within the first hour of heat exposure. In agreement with the ribosome profiling data, PsbB/CP47 and PsbC/CP43 translation showed decreased rates during early heat exposure and a recovery of translation after 60 min of acclimation (Figure 4, C). The effect of the strongly reduced synthesis of PsbB/CP47 and PsbC/CP43 was reflected in the abundance of the respective proteins in immunoblots of heat-treated cells (Figure 4, D). Protein levels of both PsbB/CP47 and PsbC/CP43 indeed decreased after the first hour of heat exposure, in line with their reduced synthesis (Figure 4, C). We also detected a modest reduction in PsbA/D1 protein levels, while TE barely changed, suggesting that PsbA/D1 might be subject to increased degradation, while the later increases of translation output and mRNA accumulation (after 60 min) may be the consequence of activated PSII repair. Importantly, PSII activity remained intact within 4 h of heat exposure at 40°C, suggesting that the observed acclimation processes contribute to successfully supply photosynthesis subunits under these stressful conditions (Figure 4, E).

### Widespread remodeling of ribosome occupancy during heat acclimation

**Figure 5:**
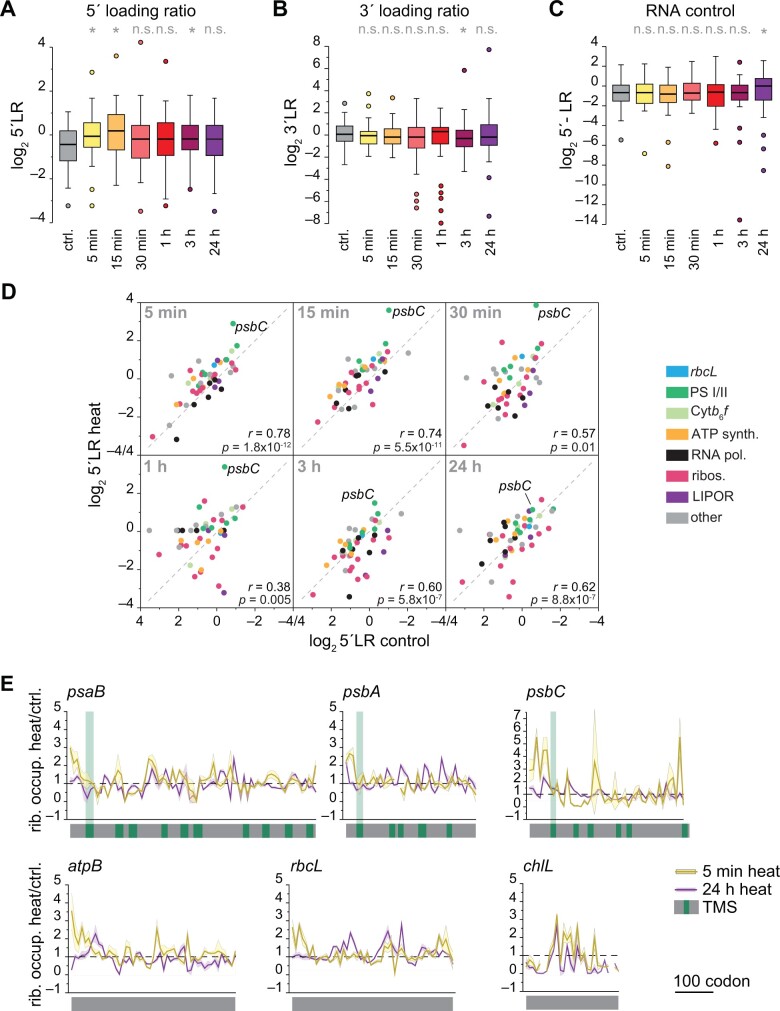
Heat exposure modifies early elongation of chloroplast translation. A, Altered 5′LR on a Log_2_ scale during early heat treatment when compared to control conditions (ctrl.). 5′LR was determined by calculating the average footprint intensities within the first six probes covering ∼60 codons of chloroplast RFs relative to the average footprint intensity of the remaining RF. Boxplots represent the distribution of all chloroplast 5′LRs. Only RFs >120 codons were examined. For each gene, the mean 5′LR was determined from three independent biological replicates. B, Control plot of ribosome footprint accumulation over the last 120 codons of a plastid RF (3′LR). C, Control plot of mRNA abundance over the first 120 codons of a plastid RF. For (A)–(C), asterisks indicate significant differences relative to the control (two-sided, paired *t*-test based *P*-values < 0.05); ns, not significant. D, 5′LRs of individual transcripts in heat-treated samples, plotted against the respective room temperature controls. Functional groups of genes are color-coded. Pearson’s correlation coefficients are given in each diagram. All values represent the mean of three biologically independent samples. E, Ratio plots over selected RFs of relative ribosome occupancy between heat-treated (5 min, dark gray; 24 h, light gray) and control samples revealing local changes of elongation. The ribosome footprint intensity was calculated as the fraction of probe signal to the summed signal for each RF and condition. The dashed line indicates the level with no change between heat and control samples. Standard deviations are plotted as ribbons calculated from three independent biological replicates. TMSs are marked in green, with the first TMS extended. Further transcripts are shown in Supplemental Figure S9.

With the obvious heat-induced changes of ribosome occupancy over *psbB* and *psbC* transcripts, we asked whether local translation changes might be detectable for all chloroplast transcripts during heat acclimation. It was clear that for most chloroplast genes, at least a fraction of the ribosome footprint intensities changed between heat-exposed samples and the control (Supplemental Figure S8). Differences in ribosome occupancy within the 5′ ends of RFs are called 5′ loading ratios (5′LRs) and were previously used to characterize heat-induced translational pausing or even stalling during translation over the first ∼60 codons per RF (Shalgi et al., 2013). Higher 5′LR values are indicative of pausing, stalling or premature termination of elongation after approximately 65 codons in mammalian cells and *Escherichia coli* (Shalgi et al., 2013; Zhang et al., 2017). Indeed, the 5′LR rose substantially for several chloroplast genes in the first 15 min of heat exposure (Figure 5, A), suggesting similar ribosomal pausing or stalling events around codon 65 in plastids. In contrast, the 3′LR in ribosome footprint samples and the 5′LR in total RNA samples showed no significant changes in the first hour of heat exposure (Figure 5, B and C). The differences in 5′LR of individual transcripts were most pronounced between 30 and 60 min of heat exposure (lowest correlation coefficients) and became smaller again during later heat exposure (Figure 5, D), presumably when acclimation reactions remodel biogenesis processes. Increased 5′LR values from heat-exposed samples were most pronounced for *psbC*, but were also noticeable, although to a lesser degree, for RFs encoding other membrane proteins (Figure 5, D). To extend such analyses to individual RFs, we plotted the ratio of ribosome occupancy between control and heat samples for exemplary RFs with transmembrane domains (Figure 5, E; Supplemental Figure S9). The resulting ratio was higher before the first transmembrane domain than the average of each RF, suggesting that any pausing or stalling might occur before the first transmembrane domain is synthesized. We also observed a higher ribosome occupancy close to the 5′ of the RF for transcripts encoding complex subunits without transmembrane domains (*atpB* and *rbcL*), but not for transcripts of noncomplex subunits such as ChlL or TufA (Figure 5, E; Supplemental Figure S9).

**Figure 6:**
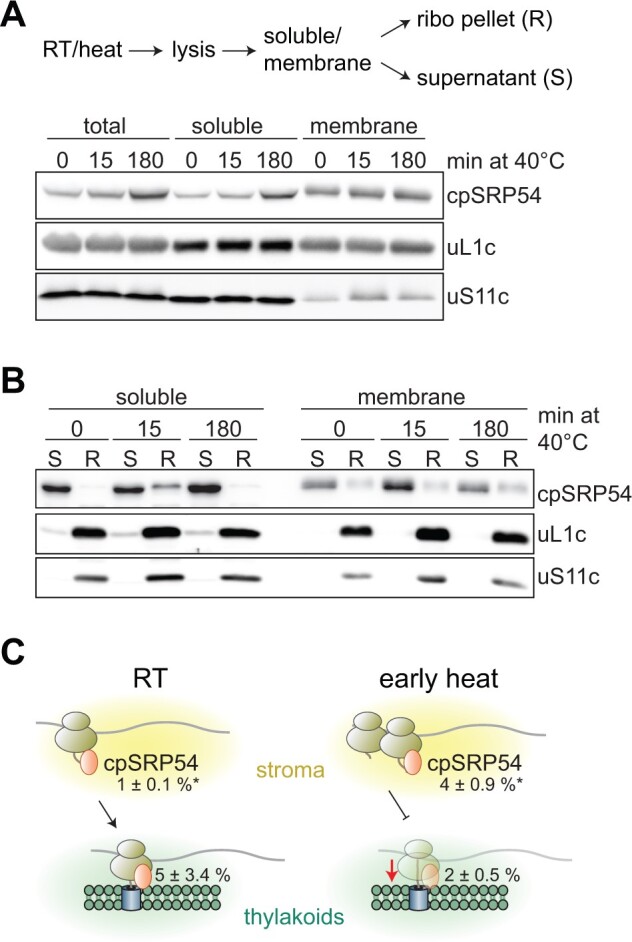
Altered ribosome-association of chloroplast SRP54 during early heat exposure. A, Cells were exposed to 40°C for 0, 15, or 180 min, harvested, and lysates were separated into soluble and membrane fractions. B, Ribosomal complexes (R) were separated from nonribosomal fractions (S) by centrifugation through a 25% (w/v) sucrose cushion. C, Schematic representation of putative targeting defects during early heat exposure. Under elevated temperatures, cpSRP54-bound ribosomes appear to accumulate in the stroma and are unable to associate efficiently with thylakoid membranes. Reduced targeting leads to an accumulation of ribosomes along the 5′ sections of transcripts encoding thylakoid membrane proteins. Numbers represent the percentage fraction of ribosome-associated cpSRP54, relative to the total amount (mean ± standard deviation). Asterisks indicate significant changes based on unpaired two-sided *t*-tests with *P*-values < 0.05.

For membrane proteins, higher 5′LRs may be a consequence of prolonged ribosome dwell time caused by insufficient membrane targeting of ribosome-nascent chain complexes during early heat treatment. We thus tested if chloroplast ribosomes were less membrane-anchored under these conditions. Accordingly, we separated proteins from control cells and cells exposed to short (15 min) and prolonged (180 min) heat into soluble and membrane fractions and analyzed them via immunoblotting. Consistent with previous reports (Chua et al., 1976), the membrane-bound ribosome pool was generally smaller than the soluble pool. However, heat exposure did not lead to significant changes in the membrane-bound ribosome pool (as judged by uL1c and uS11c; Figure 6, A). Notably, we observed that ribosome binding of cpSRP54 is altered during heat exposure. CpSRP54 is thought to be a main driver for membrane targeting of ribosomes translating nascent thylakoid proteins (Ries et al., 2020). Sedimentation of soluble or membrane-associated chloroplast ribosome fractions by ultracentrifugation showed a higher signal of ribosome-bound cpSRP54 in the soluble fraction of cells heat-treated for 15 min when compared to the control, indicating that ribosome-nascent chain complexes recruited by cpSRP54 accumulate in the early phase of heat acclimation (Figure 6, B and C). In contrast, the abundance of ribosome-bound cpSRP54 decreased in the membrane fraction after 15 min of heat exposure (Figure 6, B and C). The effect disappeared after long-term heat acclimation. Taken together, heat exposure may affect thylakoid membrane translocation, which would explain the altered ribosome-recruitment of cpSRP54 in the soluble fraction and the altered ribosome occupancy at the 5′ sequence of transcripts encoding thylakoid membrane proteins.

### Heat-mediated changes in plastid translation are conserved between Chlamydomonas and tobacco

**Figure 7:**
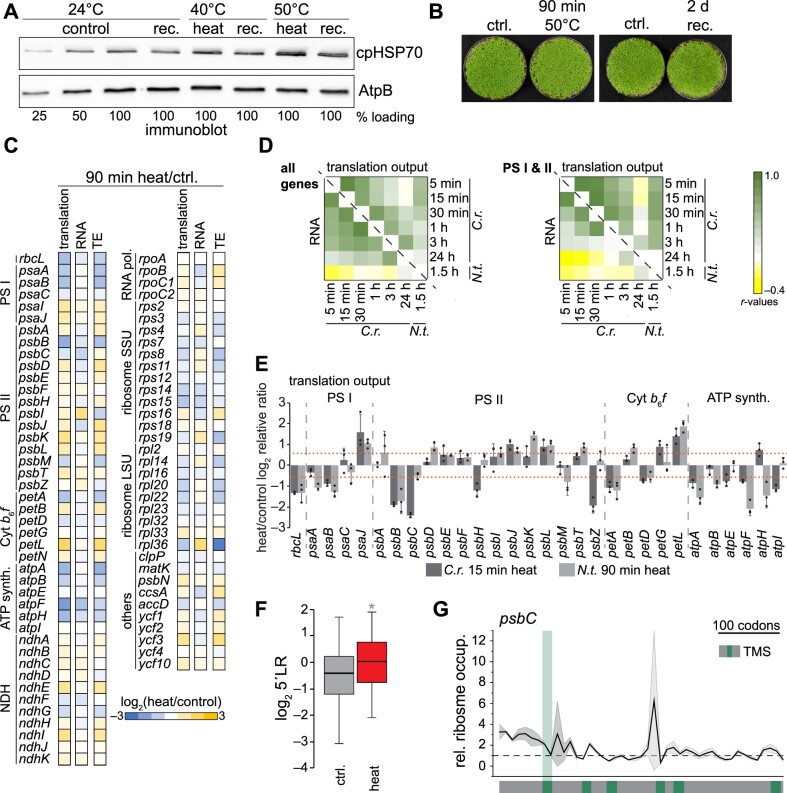
Heat-induced changes of chloroplast translation are widely conserved between a green alga and a vascular plant. Heat-induced changes of chloroplast RNA accumulation and translation output in tobacco (*N. tabacum*). A, Immunoblot analysis of heat-treated tobacco samples exposed for 90 min to 40°C or 50°C, allowed to recover for 2 days at 24°C (rec.), or remained untreated (control). Reversible heat induction of the cellular stress response is demonstrated by probing against chloroplast HSP70; AtpB serves as loading control. B, Images of tobacco seedlings exposed to heat at 50°C for 90 min. For recovery (rec.), plants were returned to 24°C for 2 days. Control plants (ctrl.) remained untreated. C, Heatmap representation of the relative values of translation output, transcript accumulation data (RNA) and TE. All values are the mean of three independent biological replicates and are plotted on a Log_2_ scale and colored as indicated in the legend at the bottom. Each row represents one gene. Respective column plots with indicated significant changes are shown in Supplemental Figure S11. D, Heatmap of Pearson’s correlation coefficients comparing heat-induced changes of translation output (above diagonal line) and RNA accumulation (below diagonal line) between Chlamydomonas heat kinetics and tobacco data. Comparison was performed for all chloroplast genes, or genes of photosystems I and II (PSI and II). E, Column plot comparing the heat-induced changes of translation output of photosynthetic genes between Chlamydomonas and tobacco. Dashed lines indicate changes >1.5-fold. All values are the mean of three independently grown replicates ± standard deviation; individual data points are shown as black circles. F, Changes of 5′LR between heat and control data of translation output. Asterisk indicates two-sided, paired *t*-test *P*-values < 0.05. G, Ratio plot for *psbC* of relative ribosome occupancy between heat-treated and control samples. The ribosome footprint intensity was calculated as the fraction of probe signal to the summed signal for each condition. The dashed line indicates the value with no change between heat and control samples. Standard deviations are plotted as ribbons, calculated from three independent biological replicates. Positions of the TMSs were extracted from https://www.uniprot.org and are marked in green, with the first TMS extended. Additional transcripts are shown in Supplemental Figure S13.

We next examined if the findings concerning heat-induced translational remodeling in Chlamydomonas were also conserved in a vascular plant species. To this end, we exposed 8-day-old tobacco (*Nicotiana tabacum*) seedlings to 40°C or 50°C heat for 90 min in a water-saturated environment (preventing additional drought stress and leaf temperature adjustment by cooling through transpiration) and monitored induction of the chloroplast-localized heat shock protein HSP70. Since the heat response induction at 50°C was stronger yet still reversible (Figure 7, A), and no tissue damage was detectable under this condition (Figure 7, B), we performed ribosome profiling in control plants and in plants exposed to 50°C. We determined translation output, RNA accumulation, and TE as earlier. As with Chlamydomonas, we detected significant changes in translation for heat-exposed tobacco seedlings with high reproducibility (Figure 7, C; Supplemental Figures S10–S12). Correlation analysis with the Chlamydomonas heat kinetics and the tobacco datasets revealed that Pearson’s correlation coefficients for translation output are highest for the early phase of the Chlamydomonas heat kinetics data, between 5 and 30 min (Figure 7, D). Generally, the heat response at the level of translation output was more conserved for transcripts encoding PSI and II subunits compared with other transcripts. In contrast, heat-induced changes in RNA levels strongly varied between Chlamydomonas and tobacco (Figure 7, D). We obtained the same result when looking at individual photosynthetic genes, for which the trend of translational control (not necessarily the exact amplitude) was conserved between 15 min heat exposure in Chlamydomonas and 90 min heat exposure in tobacco (Figure 7, E). Also consistent with the heat response in Chlamydomonas, we observed increased 5′LRs for tobacco transcripts (Figure 7, F; Supplemental Figure S13, A–C). We also detected an increased 5′LR for individual transcripts encoding thylakoid membrane proteins, as observed in Chlamydomonas (Figure 7, F–G; Supplemental Figure S13, D), although to a lower extent.

### Remodeling of the plastid ribosome interaction network during heat exposure

**Figure 8:**
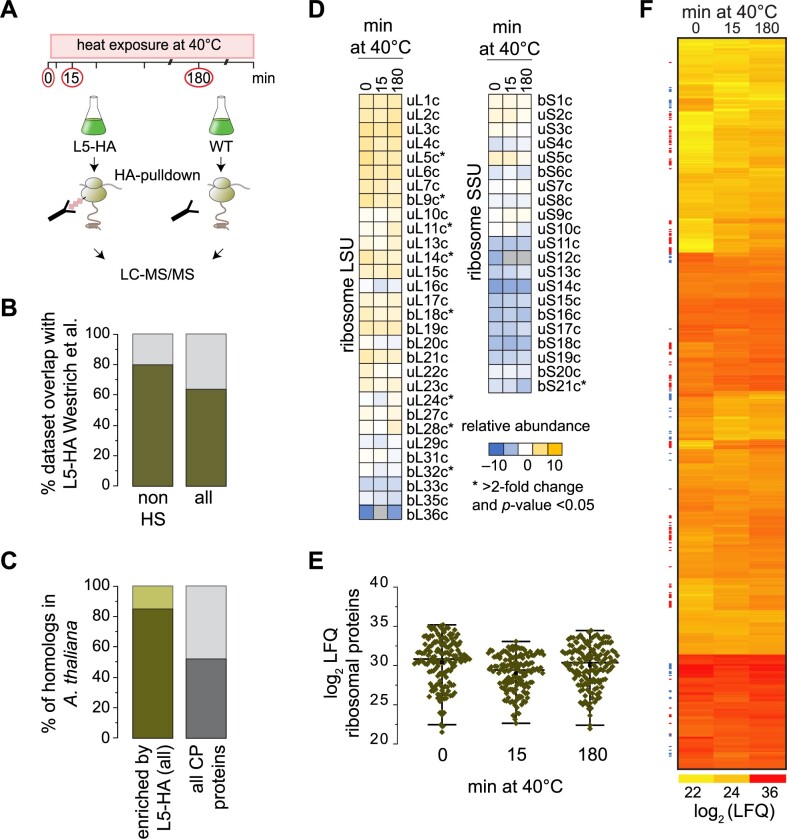
Affinity-purification MS reveals heat-induced dynamics of plastid ribosome interactors. A, Schematic diagram of the experimental setup. Cells were exposed to 40°C and harvested at the indicated time points. The control cultures (T0) were harvested after 15 min of 25°C. After AP from triple-HA tagged uL5c cells or nontagged controls, eluates were analyzed by MS. B, Overlap of proteins enriched through AP-MS with tagged-Rpl5 identified in this study (dark green) and the proteins enriched by L5-HA AP-MS from a previous study (gray; Westrich et al., 2021). Comparison is shown for the proteins only enriched under room temperature control conditions (not heat-treated, HS) and all proteins enriched in the three conditions (control, 15 min and 180 min heat, all). C, Fraction of proteins specifically enriched by L5-HA AP-MS with homologs in Arabidopsis (dark green) and Chlamydomonas proteins with a predicted chloroplast localization and whether they have homologs in Arabidopsis (gray). All homologs are listed in Supplemental Data Set S3. D, Relative abundance of chloroplast ribosomal proteins, normalized to the mean LFQ values of all measured ribosomal proteins. Ribosomal proteins with LFQ values that differ by greater than two-fold during heat exposure and with Welch’s unequal variances *t*-test *P*-values < 0.05 are marked with an asterisk. E, Distribution of LFQ values for plastid ribosomal proteins in the control experiment and after 15 or 180 min of heat exposure at 40°C. For each time point, the three replicates are drawn as separate diamonds per ribosomal protein. The black horizontal line indicates the median of the L5-HA bait LFQs of the control time point. F, Heatmap of LFQ values for all 759 proteins with annotated chloroplast localization and significantly enriched over the respective untagged control (see “Methods”). All values are the mean LFQ values from three biological replicates. Colored dots to the left of the heatmap mark proteins whose enrichment significantly changed during heat exposure (red, increased; blue, decreased abundance after 15 min heat treatment).

To gain deeper insights into the drivers that mediate remodeling of protein biogenesis during heat exposure, we purified affinity-tagged plastid ribosomes from cells and identified their interactors by subsequent mass spectrometric analyses based on our recently established protocol (Westrich et al., 2021). Prior to harvest, we crosslinked ribosomal complexes in vivo, which previously proved to be essential for enriching weakly associated proteins and to increase specificity for co-purifying mainly chloroplast-localized factors (Rohr et al., 2019; Westrich et al., 2021). We thus isolated chloroplast ribosomes from untreated Chlamydomonas cells (maintained at 25°C), or exposed to a short-term (15 min) or long-term (180 min) heat treatment. As control, we harvested untagged cells in parallel (Figure 8, A). With our highly sensitive mass spectrometer, we identified over 4,000 proteins, of which ∼2,500 remained after retaining only proteins quantified in at least two replicates for one time point (Supplemental Data Set S3). The reproducibility among biological replicates had Pearson’s correlation coefficients higher than 0.86 (Supplemental Figure S14). We tested for significant enrichment of proteins in the Rpl5-HA (L5-HA) pulldowns over untagged control via a one-sided *t*-test with a correction for multiple testing by permutation-based false discovery rate (FDR) calculation (FDR < 0.05, *S*_0_ = 1) similar to previously used parameters that allow the robust determination of ribosome enrichment (Westrich et al., 2021). Of the enriched proteins, 759 (or 85%) had a predicted or verified chloroplast localization and showed an ∼80% overlap with our previous dataset (Westrich et al., 2021; Figure 8, B). Proteins enriched in the ribosome pull-downs were more conserved in the green lineage than the average of all chloroplast proteins in Chlamydomonas (Figure 8, C), which potentially allows an extension of our conclusions to chloroplast protein biosynthesis in plants.

We first compared if the composition of the purified ribosome was altered upon heat exposure. We, therefore, calculated the relative abundance of ribosomal proteins (the ratios of each label-free quantitation (LFQ) value over average LFQ intensity of all ribosomal proteins) at each time point. While some ribosomal proteins showed altered abundance, we did not detect a major depletion of specific ribosomal proteins during heat exposure, which suggests that the ribosome itself remains overall intact during the heat treatment (Figure 8, D).

**Figure 9:**
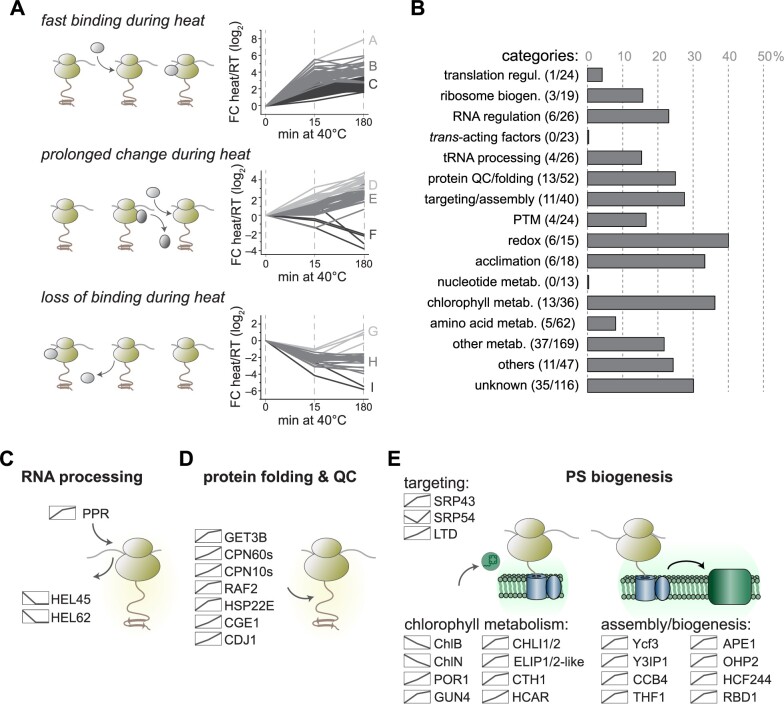
Categories of chloroplast proteins with altered ribosome association during heat acclimation. A, Trends of ribosome-associated factors whose enrichment significantly changed during heat exposure (ANOVA *P*-values < 0.05, Log_2_ LFQ were normalized to the T0 time point). Changes of fast ribosome binding (top), prolonged changes (middle) and loss of binding (bottom) upon heat treatment are indicated by the schematic diagrams. B, Changes within the categories of putative ribosome interactors. Bars indicate the fraction of factors relative to the sum of all factors in the group that changes in ribosome binding during heat exposure (numbers are given in brackets) (regul., regulation; biogen., biogenesis; PTM, posttranslational modification; metab., metabolism). C–E, Binding behavior of selected factors that show altered ribosome binding during heat treatment. The binding trend is shown as a diagram next to the protein name.

Under short-term heat conditions (15 min), LFQs of all plastidic ribosomal proteins decreased slightly compared to values for the control or long-term heat condition (Figure 8, E). We hypothesized that constant ribosome interactors should show a similar trend with reduced LFQ intensities at the 15-min time point. Cross-normalization of the three time points, based on the average intensities of the ribosomal proteins in the respective datasets, led to an artificial overrepresentation of ribosome-interacting proteins at the 15-min time point (Supplemental Figure S15). Instead, we opted to use the degree of correlation between interactors of lower abundance and the intensities of ribosomal proteins during heat exposure. We extracted a list of putative ribosome-interacting proteins with altered ribosome association during heat exposure as follows: first, we used ANOVA-testing coupled to a post-hoc test to identify inter-time point changes based on LFQ values (Figure 8, F). Second, all candidates whose abundance changes correlated with ribosomal proteins and hence might decrease due to the slightly lower levels of ribosomal bait proteins at the 15-min time point were removed from the list. Following these criteria, we identified 136 proteins with known or putative chloroplast localization that are significantly enriched on plastid ribosomes during one or both time points of heat exposure, with another 21 proteins being significantly depleted upon heat treatment (Table 1). Based on their altered ribosome association, hierarchical clustering assigned proteins into nine clusters (A–I) and three groups: fast interactors with a significant increase in ribosome binding after only 15 min of heat exposure (Clusters A–C); slow interactors with changes in ribosome binding only after 180 min of heat exposure (Clusters D–F); and interactors with lower ribosome association after 15 min of heat exposure (Clusters G–I) (Figure 9, A).

**Table 1:** Putative interactors with changes in ribosome binding during heat acclimation

Category^a^	Name^b^^,^^c^	Gene	Function^b^	Cluster^d^
Translation regulation	ArfB	Cre02.g101800	Ribosome rescue	H
Ribosome biogenesis	RH39	Cre01.g033832	RNase forming the hidden break	H
	NA	Cre17.g722250	rRNA Methyltransferase	H
	RBGA	Cre17.g747347	Ribosome Biogenesis	H
RNA regulation	RPOC2	CreCp.g006600	Plastidic RNA polymerase subunit	H
	NA	Cre01.g024251	HRB/C-type RNA helicase	H
	NA	Cre03.g213649	RNA helicase	B
	HEL45	Cre10.g436650	DEAD-box RNA helicase	H
	NA	Cre14.g611950	PPR protein	C
	HEL62	Cre16.g662000	DEAD/DEAH box helicase	H
tRNA processing	TSF2	Cre02.g079600	Phenylalanyl tRNA synthase	B
	NA	Cre08.g364650	Methionyl tRNA synthase	B
	GAT1	Cre10.g439400	Glutamyl tRNA amidotransferase subunit A	B
	CGL27	Cre13.g579550	tRNA adenosine methylthiotransferase	D
Protein QC/folding	CPN60B1	Cre17.g741450	Chaperonin	E
	CPN60B2	Cre07.g339150	Chaperonin	E
	CPN11	Cre16.g673729	Chaperonin lid	E
	CPN20	Cre08.g358562	Chaperonin lid	E
	CPN23	Cre12.g505850	Chaperonin lid	E
	RAF2	Cre01.g049000	Rubisco maturation	E
	NA	Cre02.g083065	Peptidase	B
	GET3B	Cre05.g245158	CP Sorting/Redox-activated chaperone	B
	NA	Cre07.g313122	Acylaminoacyl-peptidase	B
	CGE1	Cre07.g341600	HSP70B nucleotide exchange factor	C
	CDJ1	Cre12.g507650	HSP70B co-chaperone of	E
	NA	Cre13.g603225	moxR ATPase	E
	HSP22E	Cre14.g617450	Chaperone	B
Targeting/assembly	YCF3	CreCp.g005600	Photosystem I assembly	C
	CGL59/Y3IP1	Cre06.g280650	Photosystem I assembly	B
	RBD1	Cre07.g315150	Rubredoxin, photosystem II assembly	B
	THF1	Cre13.g562850	Photosystem II assembly	B
	CCB4	Cre08.g382300	Cytochrome *b*_6_*f* assembly	B
	CGL102/HCF244	Cre02.g142146	Photosystem II biogenesis	B
	OHP2	Cre06.g251150	Photosystem II biogenesis	C
	SRP43	Cre04.g231026	Signal recognition particle 43, sorting	C
	cpSRP54	Cre11.g479750	Signal recognition particle 54, sorting	G
	CGL38/LTD	Cre12.g551950	Sorting of imported chloroplast proteins	E
	CGLD8/PAB	Cre13.g563150	ATP synthetase assembly	B
PTM	NA	Cre06.g284650	Phosphatase	E
	NA	Cre08.g365632	Phosphatase	E
	CPKR	Cre12.g527000	Kinase	D
	RMT1	Cre16.g661350	Rubisco methyltransferase	C
Redox	PRX5	Cre01.g014350	Peroxiredoxin, type II	B
	NA	Cre02.g093650	Rieske protein, Ferredoxin related	E
	FTRB	Cre03.g193950	ferredoxin thioredoxin reductase	E
	PRX6	Cre10.g422300	Peroxiredoxin Q	E
	NA	Cre16.g687294	Ferredoxin/thioredoxin reductase subunit A	C
	NA	Cre17.g715500	Thioredoxin	B
Acclimation	CPLD42	Cre01.g004450	Light acclimation	E
	APE1	Cre16.g665250	Light acclimation, PSII association	B
	NA	Cre01.g044700	Glutathione S-transferase	B
	APX1	Cre02.g087700	Ascorbate peroxidase	B
	FLAV	Cre12.g531900	Thylakoid flavodiiron, photoprotection of PS	E
	NA	Cre15.g638400	Stress response	D
Pigment metabolism	CHLB	CreCp.g000400	Light-independent PChlide reduction	F
	CHLN	CreCp.g008000	Light-independent PChlide reduction	I
	POR1	Cre01.g015350	Light-dependent protochlorophyllide reductase	E
	DVR1	Cre01.g042800	3,8-Divinyl protochlorophyllide *a* 8-vinyl reductase	C
	GUN4	Cre05.g246800	Tetrapyrrole-binding protein	B
	CHLI1	Cre06.g306300	Magnesium chelatase subunit I, isoform 1	B
	CRD1	Cre07.g346050	Magnesium-protop. IX monomethyl ester cyclase	B
	CYP97C3	Cre08.g373100	Cytochrome P450	C
	HCAR	Cre11.g468700	7-Hydroxymethyl chlorophyll a reductase	E
	CTH1	Cre12.g510050	Mg-protoporp. IX monomethyl ester oxid. cyclase	B
	CHLI2	Cre12.g510800	Magnesium chelatase subunit I	B
	ELIP1-like	Cre13.g576760	Early light-induced protein	C
	ELIP2-like	Cre14.g626750	Early light-induced protein	C
Amino acid metabolism	AAT2	Cre06.g284700	Alanine aminotransferase	B
	HIS1	Cre09.g410650	*N*-5-phosphoribosyl-ATP transferase, His synthesis	B
	SHK5	Cre10.g436350	Shikimate kinase, amino acid synthesis	E
	HIS6	Cre12.g495300	Phosphoribosylformimino-5-aminoimidazole carboxamide ribonucleotide isomerase	B
	NA	Cre14.g627850	4-Hydroxy-tetrahydrodipicolinate reductase	B
Other metabolism	NA	Cre01.g005950	FAD/NAD(P)-binding oxidoreductase	H
	RFK2	Cre01.g025250	Riboflavin kinase	B
	TPIC1	Cre01.g029300	Triose phosphate isomerase	C
	BCC2	Cre01.g037850	Acetyl-CoA biotin carboxyl carrier	E
	NTR4	Cre01.g054150	Metabolism, NTRC	E
	NA	Cre01.g056331	Glutamine amidotransferase-like protein	B
	ZEP1	Cre02.g082550	Zeaxanthin epoxidase	C
	NA	Cre02.g143000	Glycerol-3-phosphate acyltransferase,	B
	ISA1	Cre03.g155001	Isoamylase, starch debranching enzyme	E
	NA	Cre03.g183300	Pseudoglucan	D
	SSS2	Cre03.g185250	Starch metabolism	E
	SBP1	Cre03.g185550	Calvin cycle	E
	RPI1	Cre03.g187450	Ribose-5-phosphate isomerase	B
	ISA3	Cre03.g207713	Isoamylase, starch debranching enzyme	B
	OPR2	Cre03.g210513	12-Oxophytodienoic acid reductase	A
	SSS1	Cre04.g215150	Starch metabolism	E
	NA	Cre05.g234651	Aldose 1-epimerase	H
	NA	Cre06.g278148	Glyoxylate/succinic semialdehyde reductase	F
	SBE1	Cre06.g289850	Starch branching enzyme	D
	HYDEF	Cre06.g296700	Hydrogenase	H
	NA	Cre06.g300700	Flavin reductase	B
	ZDS1	Cre07.g314150	Zeta-carotene desaturase	B
	GWD2	Cre07.g332300	R1 Protein, alpha-glucan water dikinase	F
	HEM2	Cre07.g339750	Ferrochelatase	C
	AMA3	Cre08.g384750	Alpha-amylase	E
	NA	Cre09.g387050	Putative fructokinase	D
	MDH5	Cre09.g410700	NADP-malate dehydrogenase	B
	NA	Cre10.g424100	Pyrophosphatase	E
	PDS1	Cre12.g509650	Secondary metabolism	C
	GLN3	Cre12.g530650	Glutamine synthase	B
	DXR1	Cre12.g546050	1-Deoxy-d-xylulose 5-phosphate reductoisomerase	B
	NA	Cre13.g573250	Thiosulfate sulfurtransferase	E
	NA	Cre14.g620350	Riboflavin biosynthesis	B
	VTE3	Cre14.g625450	MPBQ/MSBQ methyltransferase	D
	TAL2	Cre14.g630847	Putative transaldolase	B
	NA	Cre16.g677450	Glucose-6-phosphate 1-epimerase	B
	NA	Cre17.g744997	FAD dependent oxidoreductase, photosynthesis	H
Others	CPLD20	Cre01.g000900	FAD/NAD(P)-binding oxidoreductase	C
	NA	Cre01.g003550	Rhodanese-like protein	D
	MSH7	Cre01.g014800	DNA repair, MutS homolog	G
	TEF4	Cre02.g111450	Rhodanese-like protein	E
	FTSZ1	Cre02.g118600	Chloroplast division	C
	FTSZ2	Cre02.g142186	Chloroplast division	B
	PCC1	Cre05.g248600	Putative copper chaperone	C
	LHCA9	Cre07.g344950	Photosynthesis antenna	B
	TEF5	Cre09.g411200	Rieske [2Fe-2S] domain	E
	PSAG	Cre12.g560950	Photosystem I	C
	NA	Cre14.g616600	Thylakoid organization	B
	PSAK	Cre17.g724300	Photosystem I	C

a Proteins of unknown function are not listed.

b Name and functional annotation were retrieved from the recent genome annotation (https://phytozome-next.jgi.doe.gov/report/gene/Creinhardtii_v5_6) or based on known annotation of homologous proteins in Arabidopsis and bacteria.

c NA, no protein name exists in the annotation.

d For cluster description, see Figure  9, A and “Methods.”

For complete list, see Supplemental Data Set S3.

While proteins involved in general translation control were largely unchanged between the conditions tested here, the functional categories with the largest changes within their respective groups were RNA regulation (23% change), protein quality control (QC; 25% change), protein targeting and complex assembly (28% change), redox control (40% change), acclimation (33% change), and chlorophyll metabolism (36% change) (Figure 9, B). Many interactors belonging to the same category were also within a common cluster (Table 1). For example, proteins involved in RNA processing and components of the light-independent POR showed lower ribosome interaction during heat exposure, while proteins involved in protein folding, QC, targeting, thylakoid assembly and light-dependent chlorophyll biosynthesis exhibited higher binding (Figure 9, C–E). Interestingly, cpSRP54 displayed a characteristic decrease in ribosome binding after 15 min of heat exposure, followed by a recovery during long-term heat (Figure 9, E), which was consistent with the data from the ribosome co-sedimentation assays (Figure 6, B, note that the pulldown includes soluble and membrane proteins).

**Figure 10:**
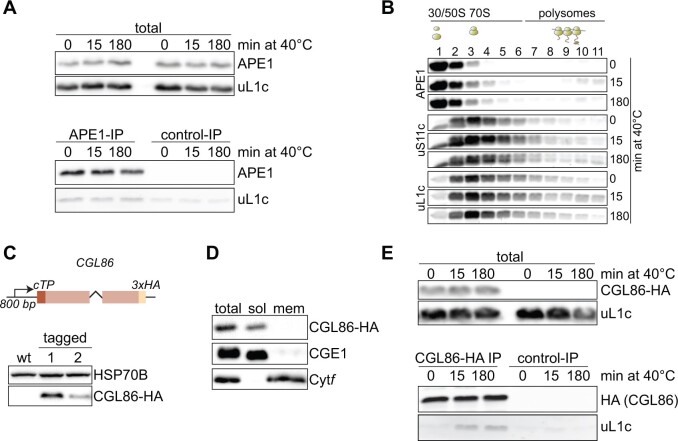
Validation of increased chloroplast ribosome interaction for APE1 and CGL86 during heat exposure. A, Co-precipitation of plastid ribosomes with APE1. Samples control and heat-treated cells were immunoblotted with antibodies against APE1 and uL1c (total). These cell lysates were used for IP with anti-APE1 antibodies (APE1-IP) or nonspecific IgGs (control-IP), and co-elution of uL1c was probed as indicated. B, Co-migration of APE1 with polysomes during heat exposure. Polysome analyses were performed as in Figure 1, E. Expected positions of unassembled ribosomal subunits, monosomes and polysomes in the gradient are marked by diagrams above the blots. C, C-terminal tagging of CGL86. Top panel: Construct generated for triple HA-tagged CGL86 under the control of the *CGL86* promoter. The first intron of *RBCS2* was inserted between the third and fourth exons. Lower panel: immunoblot of untagged (WT) and *CGL86-HA* expressing strains. HSP70B serves as loading control. D, Crude fractionation of Chlamydomonas cells. Lysates (total), soluble fraction (sol) and detergent-solubilized membranes (mem) were immunoblotted against tagged CGL86, CGE1 (for stromal proteins), and Cyt*f* (for thylakoid membrane proteins). E, Co-precipitation of plastid ribosomes with CGL86 during heat exposure. Experiments were performed as in (A) and IPs were performed with HA-decorated magnetic beads from CGL86-HA tagged and untagged (control IP) samples. All immunoblots are representative of three biological replicates from independently grown cultures.

We further validated two proteins with altered ribosome binding during heat exposure, Acclimation of Photosynthesis to the environment 1 (APE1) and Conserved in the Green Lineage 86 (CGL86). APE1 belonged to cluster B, with a maximum four-fold enrichment in ribosome binding during heat versus room temperature (Figure 9, E; Supplemental Data Set S3). APE1 has been shown to be required for high light acclimation of PSII (Walters et al., 2003; Chazaux et al., 2020). To validate its increased ribosome binding under heat, we performed immunoprecipitation (IP) assays with an anti-APE1 specific antibody (Supplemental Figure S16, A). APE1 IPs from detergent-solubilized cells revealed co-elution of the ribosomal protein uL1c with APE1, whereas control IPs showed only traces of nonspecifically bound uL1c (Figure 10, A). Moreover, slightly more APE1 co-sedimented with ribosomes of the membrane fraction during early heat exposure (15 min; Supplemental Figure S16, B). Since ribosome association of APE1 only marginally increased during heat acclimation, we further looked at altered co-migration of APE1 with translating plastid ribosomes in sucrose density gradients. Under ambient conditions, we detected no APE1 in polysome fractions, while heat exposure led to a visible co-migration of APE1 with polysomes (fractions 7–11; Figure 10, B), again pointing to the increased association of APE1 with ribosomes during heat acclimation.

One of the most strongly enriched factors (16-fold increased ribosome binding under heat versus ambient conditions; Supplemental Data Set S3) was the uncharacterized protein CGL86 (Cre12.g534450), which harbors a predicted N-terminal chloroplast transit peptide (cTP) resulting in a 14.4-kD mature protein. CGL86 also contained a highly conserved forkhead-like domain, which may point to an involvement in mRNA processing (Yu et al., 2008). HA-tagged CGL86 mainly accumulated in the soluble fraction of Chlamydomonas cells and did not co-precipitate with ribosomal proteins under ambient conditions. Importantly, heat led to its clear co-elution with ribosomal protein uL1c (Figure 10, C–E), hence confirming our affinity purification-mass spectrometry (AP-MS) findings.

## Discussion

Chloroplasts are central organelles that sense various environmental stimuli and integrate them into subcellular acclimation reactions (Kleine et al., 2020). We uncovered evidence that the translation output of subunits of the photosynthetic machinery is differentially affected during acclimation to high temperature, that the pattern of translational changes is conserved between Chlamydomonas and tobacco for photosynthetic genes, and that proteins involved in nascent chain folding, maturation, co-factor attachment and complex assembly increasingly interact with ribosomes during heat acclimation.

### General dynamics of plastid translation during heat acclimation

Consistent with previous data (Uniacke and Zerges, 2008), we failed to observe formation of temperature-induced stress granules, which are known to sequester ribosome-mRNA complexes in the cytosol during stress (Mahboubi and Stochaj, 2017). While heat exposure did not lead to a global arrest of chloroplast translation (Figure 1, D–E), it did alter the subcellular localization of chloroplast ribosomes from a rather even distribution to foci adjacent to the pyrenoid (Figure 1, A–C). These basal regions have been previously identified as major biogenesis sites and thus been termed “translation zones” (T-zones) (Uniacke and Zerges, 2007; Sun et al., 2019). The chloroplast envelope membranes adjacent to the T-zones were reported to be the sites of protein import for nucleus-encoded chloroplast proteins (Schottkowski et al., 2012). Hence, a relocation of chloroplast protein synthesis to these T-zones under unfavorable conditions would minimize diffusion distances between newly synthesized chloroplast- and nucleus-encoded subunits of thylakoid protein complexes. Heat also affected ribosome dynamics considerably in both Chlamydomonas and tobacco (Supplemental Figures S8, S12), while high light was previously reported to have only mild effects (Schuster et al., 2019). Interestingly, most transcripts encoding thylakoid membrane proteins showed an increased ribosome accumulation within their first 60–100 codons (5′LR) during the same time of heat exposure (Figures 5, 7). Such increased ribosome accumulation over the first 60 codons was also observed for cytosolic translation during heat exposure and proteotoxic stress in mammalian and bacterial cells (Liu et al., 2013; Shalgi et al., 2013; Zhang et al., 2017). In these earlier studies, the observed pile up of ribosomes at the initiation codon and the first 60 codons of the RF during heat stress were directly attributed to the loss of Hsp70 binding to translating ribosomes, since nascent chain folding by Hsp70 is required for fast translation. However, we did not observe altered ribosome binding of the single chloroplast Hsp70, HSP70B, in our AP-MS dataset (Supplemental Data Set S3). Instead, the smaller pool of membrane-associated cpSRP54 (Figure 6) and the increased footprint density upstream of the mRNA segment encoding the first transmembrane domain segment (TMS; Figure 5, E) point to an altered process of co-translational targeting of nascent membrane proteins into thylakoids. Additionally, the increased 5′LRs induced by heat for complex subunits that are soluble or not directly integrated into thylakoid membranes (i.e. RbcL and AtpB; Figure 5, E; Supplemental Figure S9) opens the possibility that plastid complexes might be, at least partially, co-translationally assembled, as observed for cytosolic complexes, and that these assembly cascades are retarded or temporally stalled during heat exposure (Shiber et al., 2018; Kramer et al., 2019).

### Effect of temperature on synthesis of photosynthesis proteins

The most evident effect of heat on translation output was the strong reduction for *psbB* and *psbC* in the first hour of heat treatment. Pulse labeling confirmed that the observed reduction of ribosome occupancy leads to lower translation rates of these two proteins during early heat exposure (Figure 4, C). While ribosome occupancy was lower over the entire RF of *psbB*, it only dropped after the first 60 codons of *psbC* (Figure 4, A and B), suggesting a different mechanism of translational reduction for synthesis of these two PSII antenna proteins. The heat-caused drop of ribosome occupancy beyond ∼60 codons for *psbC* points to elongational arrest. In contrast, *psbB* translation might be downregulated at the level of initiation. In Chlamydomonas, the *psbB* 5′ untranslated region (5′ UTR) leader sequence is remarkably short (Cavaiuolo et al., 2017; Gallaher et al., 2018) and the *psbB* mRNA maturation factor, MBB1, is the only known trans*-*acting factor that promotes expression of algal *psbB* (Vaistij et al., 2000). At this point, we cannot exclude a regulatory function for MBB1 during heat, or for other as yet to be described trans*-*factor(s) that may control heat-induced alterations in translation. Despite the presence of several trans-factors in the ribosome interactome, we identified no putative trans-factor such as an uncharacterized octotricopeptide repeat protein with differential ribosome binding during heat (Figure 9, C). Alternatively, the lower translation of *psbB* might be the result of the known effect in the tightly controlled assembly cascade of PSII that causes translational autoinhibition of *psbB* by unassembled PsbB/CP47 proteins (Minai et al., 2006).

The fast translational decrease seen for PsbB/CP47 and PsbC/CP43 synthesis is intriguing, since the other two core proteins of PSII, namely PsbA/D1 and PsbD/D2, were largely unaffected during early heat exposure (Figure 3, A). Translation output of PsbA/D1, PsbD/D2, and also Cyt*b*_559_ (containing subunits PsbE and PsbF) gradually increased during the heat kinetics (Supplemental Figure S4). These components might be continually renewed throughout heat acclimation to replace damaged and degraded PsbA/D1, PsbD/D2, and Cyt*b*_559_ in existing PSII core complexes. The mild increase of PsbA/D1 synthesis is apparently sufficient to compensate for the increased turnover caused by heat damage and for maintaining photosynthetic activity (Figure 4, D and E). Importantly, the adjusted translation output of transcripts encoding PSI and PSII subunits followed the same trend under heat in Chlamydomonas and tobacco, suggesting conserved mechanisms of producing these subunits during heat acclimation (Figure 7, C), consistent with our previous observation that translation output is highly comparable between Chlamydomonas and land plants (Trösch et al., 2018).

### Factors involved in complex assembly are enriched at ribosomes during heat

The comparison of plastid ribosome-associated factors during ambient conditions after 15 and 180 min of heat exposure by AP-MS revealed 157 proteins with differential ribosome association during the heat treatment (Supplemental Data Set S3). The functional categories with the most striking changes in ribosome association included protein targeting, translocation, and assembly of thylakoid membrane proteins (Figure 9, B). While ribosome association of the co-translationally acting trigger factor chaperone, TIG1 (Ries et al., 2017; Rohr et al., 2019), did not change, cpSRP54 abundance at the ribosome decreased approximately two-fold after 15 min of heat exposure and recovered to control levels after 180 min of heat (Figure 9, E). Interestingly, we measured enrichment of two factors involved in targeting of imported nucleus-encoded thylakoid proteins, cpSRP43 and the light-harvesting chlorophyll- and carotenoid-binding protein (LHCP) translocation defect protein LTD (Figure 9, E). Both factors are not reported to bind chloroplast ribosomes, which is consistent with their absence in the L5-HA dataset under control conditions (Supplemental Data Set S3) (Olinares et al., 2010; Westrich et al., 2021). It is noteworthy that we also specifically enriched for proteins at ribosomes under heat but not under control conditions that act further downstream during assembly of thylakoid complexes involved in photosynthesis (Figure 9, E). For example, the PSI assembly proteins Ycf3 and the Ycf3-interacting protein Y3IP1 were previously shown to form an essential module for the assembly of nascent PsaA and PsaB into a reaction center heterodimer (NellaepalLi et al., 2018) and were enriched co-translationally upon heat (Figure 9, E). Overexpression of *Y3IP1* in Arabidopsis leads to plants with higher tolerance to several abiotic stressors including heat stress, suggesting that co-translational action of Y3IP1 might be important during acclimation. For PSII, an intriguing factor that is enriched on ribosomes during heat exposure is APE1, which was identified from a screen for Arabidopsis mutants defective in light acclimation, as its absence causes slight defects of PSII during high light transition (Walters et al., 2003). Recent findings proposed that APE1 binds to the PSII core complex in Chlamydomonas (Chazaux et al., 2020). Various approaches confirmed the increased ribosome association of APE1 during heat (Figure 10), indicating that APE1 might be a PSII biogenesis factor with a particular function for PSII biogenesis during stress conditions.

Interestingly, we observed significantly increased ribosome association during heat for enzymes involved in light-dependent chlorophyll biosynthesis, while the light-independent POR enzymes ChlB and ChlN displayed reduced binding during heat exposure (Figure 9, E). Several of these enzymes were previously enriched by chloroplast ribosome AP from Chlamydomonas cells (Westrich et al., 2021). This is an intriguing result, since heat affects the synthesis of PsbB/CP47 and PsbC/CP43, the main chlorophyll *a* binding proteins in the PSII core complex (Caffarri et al., 2014). Consistently, PsaA and PsaB, the main chlorophyll *a* binding proteins of the PSI complex, were also translationally reduced (greater than two-fold and greater than three-fold reduction of TE, respectively, Supplemental Figure S3). Hence, this reduction might be the consequence of insufficient chlorophyll availability during translation under heat. Such an explanation is consistent with previous reports about reduced chlorophyll synthesis, aggregation of chlorophyll biogenesis enzymes, or increased chlorophyll degradation during heat exposure (Mathur et al., 2014; Rütgers et al., 2017). The tight control of pigment accumulation is essential, since free chlorophyll during repair of chlorophyll-binding proteins would lead to an accumulation of reactive oxygen species causing critical damage to proteins and lipids. One mechanism for rescuing the biosynthesis of chlorophyll *a* under heat may entail the gain of co-translationally acting 7-hydroxymethyl chlorophyll *a* reductase, HCAR observed in this study, as it catalyzes the rate-limiting step in the conversion of chlorophyll *b* to *a* (Meguro et al., 2011)*.* Finally, homologs of the Arabidopsis Early Light-Inducible Proteins ELIP1 and ELIP2 are associated with ribosomes only under heat conditions (Figure 9, E; Table 1). ELIPs transiently bind free chlorophylls (especially chlorophyll *a*) and serve as pigment carriers during the assembly of photosynthesis complexes (Adamska et al., 1999; Montane and Kloppstech, 2000; Casazza et al., 2005). Importantly, many factors that show increased ribosome interaction during heat acclimation such as APE1, Ycf3, or CTH1B do not generally accumulate during heat exposure (Hemme et al., 2014), arguing against an assumption that increased ribosome association is just a consequence of increased protein abundance. In sum, heat transiently challenges the biogenesis of chlorophyll *a*-containing PS subunits. Acclimation and subsequent relief from these primary translational effects include chlorophyll *a* salvage and enhanced co-translational action of assembly factors. The exact mechanistic details of these co-translational processes during heat acclimation, however, need to be examined in more detail in future studies.

## Materials and methods

### Algal and plant material

For the heat kinetics experiment, *C.* *reinhardtii* strain CC-1690 was grown mixotrophically in Tris Acetate Phosphate (TAP) medium (Kropat et al., 2011) on a rotary shaker at 25°C and an illumination of 80 µmol m^−2^ s^−1^ (MASTER LEDtube HF 1,200 mm UO 16W830 T8 and 16W840 T8, Philips). The cells were harvested in mid-logarithmic phase. For ribosome AP-MS pulldown experiments, chloroplast-encoded uL5c ribosomal protein of *Chlamydomonas* CC-1690 strains was tagged by cloning the sequence for a triple HA-tag in-frame and downstream of the *rpl5* coding sequence (CDS) in the plasmid pUCatpXaadA (Goldschmidt-Clermont, 1991) to generate the construct pFW182. For details about cloning, screening and validation, see Westrich et al. (2021). All replicates of Chlamydomonas cells were grown for several days in separate flasks under the same growth conditions and started from different precultures.

Wild-type tobacco (*N.* *tabacum*, cv. Petit Havana) was germinated on a nylon net over water-saturated vermiculite in plates and grown for 8 d under controlled conditions (16-h light/8-h dark, 350 µmol m^−2^ s^−1^, 24°C/20°C) in Conviron BDW80 growth chambers with Roschwege LED lights. Five hours after lights-on, half of the plates were heated for 90 min to 50°C in a water bath, while the remaining plates were kept at 24°C as control samples (both samples were kept in the same light conditions). Heat-treated and control samples were harvested, snap-frozen in liquid nitrogen, and stored at –80°C until further use. Biological replicates were grown in separate plates under the same growth conditions.

### Kinetics of heat exposure

For Chlamydomonas, cells were grown to mid-logarithmic phase (3–4 × 10^6^ cells mL^−1^) and then centrifuged at room temperature at 3,000 *g* for 2 min. The cell pellets were resuspended either in prewarmed, 40°C TAP medium (heat kinetic) or in TAP medium equilibrated to room temperature or 25°C (control). Heat-exposed cultures were agitated in a 40°C water bath and control cultures were placed on a rotary shaker next to the water bath under the same light source. For each time point of the kinetic analysis, 8 mL of culture was harvested for total RNA extraction; the cells were pelleted by centrifugation at room temperature at 3,000 *g* for 2 min, and the pellet was immediately resuspended in 750 µL Trizol. For footprint extraction, samples were supplemented with 100 µg mL^−1^ chloramphenicol and 100 µg mL^−1^ cycloheximide and immediately cooled down to <8°C with plastic ice cubes. Cells were then pelleted at 4°C at 3,000 *g* for 2 min, the pellets were washed once with ice-cold polysome buffer (20 mM Tris–HCl pH 8.0, 25 mM KCl, 25 mM MgCl_2_, 1 mM dithiothreitol (DTT), 100 µg mL^−1^ chloramphenicol, and 100 µg mL^−1^ cycloheximide) and then flash-frozen in liquid nitrogen. For Chlamydomonas and tobacco samples, ribosome footprint and total RNA extraction and their preparation for high-resolution tiling microarray analysis were described before (Trösch et al., 2018). All biological replicates were processed in parallel.

### RNA labeling, microarray, and data analysis

Microarray hybridization and data analysis were performed as described before (Trösch et al., 2018). In brief, signal intensities from mRNA and ribosome footprint samples were extracted for all probes covering all annotated and confirmed RFs and normalized to the mean signal of datasets. Average values for each RF were determined from normalized data per replicate. Relative abundance of translation output and RNA abundance were determined by normalizing each Log_2_-transformed average value to the average RF signal per dataset. Relative TE was determined by calculating the ratio of translation output over mRNA abundance. All mean values and standard deviations of translation output, mRNA abundance and TE were determined from three independent biological samples. Ratios between heat-treated and the respective control sample were calculated for the relative translation output, mRNA and TE data. Significances of gene-specific changes between heat-treated and control samples were determined with a Welch's unequal variances *t*-test and corrected for multiple testing according to the Storey’s *q*-value method. Only genes with a *q*-value ≤ 0.05 and expression changes over 1.5-fold were marked as significant. For calculation of the 5′LR, only genes that were covered with at least six probes and had ˂50% missing values were considered. Ribosome footprint signal intensities of the six probes at the beginning of a respective RF were averaged and divided by the average probe values of the remaining RF. 5′LR values are the mean values of three independent biological replicates. Significant differences between control and heat samples were determined with a two-sided paired *t*-test and indicated with asterisks for *P*-values ≤ 0.05. PCAs, ANOVA-testing (permutation-based FDR calculation; *q*-values ≤ 0.05; *s* = 1), and hierarchical cluster pattern analysis was performed with *Perseus* software version 1.6.3.2 (Tyanova et al., 2016).

### Ribosome pulldown analysis

Untagged CC-1690 and *uL5c-HA* strains were grown to early logarithmic phase (2 × 10^6^ cells mL^−1^) in HEPES, acetate, phosphate (HAP) medium (Westrich et al., 2021), harvested by centrifugation at 4,000 *g* at 23°C for 2 min and resuspended in HAP prewarmed to 40°C or 25°C-HAP for the control. Heat was applied for 15 min and 3 h, respectively, while the control remained at 25°C and was harvested after 15 min. After the respective incubation time, formaldehyde was added for 10 min at 0.37% (w/v) final concentration for in vivo crosslinking. Crosslinking was quenched for 5 min by the addition of 100 mM Tris–HCl pH 8.0, 100 µg mL^−1^ chloramphenicol, and 100 µg mL^−1^ cycloheximide. After incubation, cells were cooled rapidly using plastic ice cubes and harvested by centrifugation. Ribosome AP followed by MS of the eluates was described before (Westrich et al., 2021). Samples of each replicate were extracted on separate days.

MS data were processed by MaxQuant version 1.6.3.3 using default parameters (Cox et al., 2014) and MS/MS spectra were searched against the Chlamydomonas database downloaded from Phytozome. The data were concatenated with reverse copies of all sequences and a list of amino acid sequences of frequently observed contaminants (minimal peptide length = 7, minimal peptide = 2 [razor or unique], PSM FDR = 0.01). For label-free quantification, the minimal ratio count was set to 2 and “match between runs” was performed with a time window = 0.7 min and an alignment time window = 20 min. All MS raw files, MaxQuant results and parameter files are accessible via ProteomeXchange. Data were processed with the *Perseus* software (Tyanova et al., 2016). All contaminant proteins, peptides identified with reverse sequences or with modifications were excluded, and missing values were imputed from a normal distribution with a downshift of 1.5× standard deviations of the real values and a width of 0.5 standard deviations after proteins were removed that were identified ˂2 times at one timepoint (Westrich et al., 2021). Similar to our previous study, the enrichment of proteins in the *uL5c-HA* dataset was determined based on Log_2_-transformed LFQ-values with a one-sided *t*-test, permutation-based FDR of 0.05 and *S*_0_ = 1 (Tusher et al., 2001). Inter-time point changes were determined by a one-way ANOVA test applying the same truncation parameters as before. Ribosome interactors with significantly altered abundances during the heat treatment were extracted by a post-hoc test, also revealing the time points of altered binding. To remove biases of varying bait amounts (different abundances of LSU ribosomal proteins during the time course) between the different time points, an average profile from all LSU-ribosomal proteins was calculated. Only those proteins were considered to exhibit heat-mediated changes of ribosome association that did not correlate with the pattern of the average LSU values (Pearson’s distance calculation with an FDR < 0.05). Proteins with no obvious predicted or experimentally determined chloroplast localization were removed. Remaining proteins were manually classified into three categories to facilitate interpretation of the data. Proteins with significantly changed abundance after 15 min of heat exposure were divided into the two groups “fast binding” and “loss of binding”, with the remaining fraction forming the group “prolonged change”. These groups were subjected to hierarchical clustering with complete linkage.

### Immunofluorescence microscopy and FISH

Immunofluorescence was performed as described before (Ries et al., 2017). For FISH, cells were grown to a density of 1–3 × 10^6^ cells mL^−1^, pelleted, and resuspended in prewarmed TAP at 40°C or TAP at control conditions (see above). Heat-treated cultures were incubated for 15, 30, and 60 min at 40°C, the control for 30 min at 25°C. After incubation, cells were fixed with 4% (w/v) formaldehyde for 1 h at 4°C and spotted onto diagnostic microscope slides (Thermo Fisher Scientific, Waltham, MA, USA) coated with 0.1% (w/v) poly-(l)-Lysine. Cells were allowed to settle, and chlorophyll was removed for 10 min with methanol at –20°C. The slides were washed with phosphate-buffered saline (PBS) and permeabilized with 2% (v/v) Triton X-100 in PBS. After washes with PBS (including 5 mM MgCl_2_), 10 ng target mRNA (EubI, EubII, and EubIII labeled with Cy5) (Daims et al., 1999) was diluted in hybridization buffer (900 mM NaCl, 20 mM Tris–HCl pH 7.5, 20% [w/v] formamide and 0.025% [w/v] SDS), added onto each spot and incubated at 46°C for 90 min in a humidity chamber filled with hybridization buffer. Afterwards, the slides were incubated for 15 min in washing buffer (100 mM NaCl, 20 mM Tris–HCl pH 7.5, 5 mM EDTA, and 0.01% [w/v] SDS), dried and blocked with 1% (w/v) BSA in PBS (PBS–BSA) for 30 min. Antibodies against uL1c and HSP22E/F were diluted 1:2,000 in PBS–BSA and incubated overnight at 4°C. The secondary fluorescein isothiocyanate (FITC)-labeled antibody (Invitrogen, Thermo Fisher Scientific, Waltham, MA, USA) was applied in a 1:200 dilution and incubated for 90 min at 25°C. Then, the slides were washed in PBS, and a drop of mounting solution containing DAPI (Vectashield; Vector Laboratories, San Francisco, CA, USA) was applied at the center of each spot. Images were acquired using a Zeiss LSM880 AxioObserver confocal laser scanning microscope equipped with a Zeiss C-Apochromat 40×/1,2 W AutoCorr M27 water-immersion objective. Fluorescent signals of FITC (excitation/emission 488 nm/493–568 nm) and tetramethylrhodamine (excitation/emission 543 nm/553–669 nm) were processed using the Zeiss software ZEN 2.3 or Fiji software. For double labeling, images were acquired using sequential scan mode to avoid channel crosstalk. Signal quantification at the T-zones was conducted with Fiji software. For each cell, two areas adjacent to the pyrenoid where boxed and the mean signal intensity was calculated, this signal was then normalized to the mean signal intensity of the whole cell.

### Cell fractionation and ribosome co-sedimentation

Chlamydomonas cells (CC-1690) were grown and subjected to heat treatment under the same conditions as for the pull-down experiments, without crosslinking. After harvesting, cells were frozen in droplets with lysis buffer (50 mM Tris pH 8.0, 25 mM KCl, 10 mM MgCl_2_, 1 mM dithiothreitol, 100 µg mL^−1^ chloramphenicol, 100 µg mL^−1^ cycloheximide, 0.2 mg mL^−1^ heparin and 50 µL mL^−1^ ribosyl-vanadyl complex) in liquid nitrogen and lysed by cryogenic milling twice at 27 Hz for 2 min. Soluble lysate was separated from membrane proteins by centrifugation for 15 min at 16,000 *g* at 4°C: The membrane pellet was washed in lysis buffer and then homogenized in fresh lysis buffer containing 1% (w/v) dodecyl-β-maltoside for 20 min and centrifuged again. Equal fractions were analyzed by immunoblotting. For the co-sedimentation assay, soluble and membrane fractions were taken from the preparation above and were loaded on a 25% (w/v) sucrose cushion and centrifuged as before (Rohr et al., 2019). The ribosomal pellet was resuspended in lysis buffer, denatured and analyzed by immunoblotting. Biological replicates were processed independently.

### Photosynthetic measurements

Chlamydomonas CC-1690 strain was mixotrophically grown to mid-logarithmic phase under constant light (60 µmol m^−2^ s^−1^) and shaking (120 rpm) at 25°C. Cells were harvested at 4,000 *g* at 21°C for 2 min and resuspended in prewarmed (40°C) TAP medium. Controls were harvested and resuspended in TAP medium at 25°C. Cultures were then transferred to a 40°C water bath, control cultures were kept at 25°C, both shaking at 120 rpm and under 60 µmol m^−2^ s^−1^. Maximum quantum yield of PSII (*Fv/Fm*) was measured with a MINI-PAM-II Photosynthesis Yield Analyzer from Walz. Fluorescence levels were measured during 3 min of dark adaptation in the cuvette, and during subsequent excitation with a saturated pulse. Biological replicates were grown in separate flasks under the same growth conditions and measured independently.

### Pulse labeling

Mid-logarithmically growing CC-1690 Chlamydomonas cells were centrifuged at 4,000 *g* at 21°C for 4 min, washed with Tris Phosphate (TP) medium (lacking acetate) and resuspended in fresh TP medium (2 × 10^8^ cells per sample). Cells were allowed to be depleted of their intracellular carbon pool for 90 min under strong agitation on a rotary shaker at 300 rpm and an illumination of 20 µmol m^−2^ s^−1^ at 25°C. Cells were pulse-labeled prior to centrifugation as a first control. For heat treatment, cells were centrifuged at 3,000 rpm and resuspended in 40°C prewarmed TP medium, pulse-labeled at time points 0, 15, 60, and 180 min of heat exposure in a growth chamber at 40°C. As a second control, cells were resuspended in TP medium at 25°C and pulse-labeled at the same time points. Pulse labeling was achieved by the addition of 10 µCi mL^−1^ Na-[^14^C]-acetate (Perkin Elmer: 56.6 mCi mM^−1^) and 10 µM mL^−1^ cycloheximide for 7 min. Labeling was stopped by transferring cells into 35 mL of ice-cold TAP medium including 50 mM nonradioactive acetate. Cells were harvested and resuspended in ice-cold 0.1 M DTT and 0.1 M Na_2_CO_3_ prior to sodium dodecyl sulphate–polyacrylamide gel electrophoresis (SDS–PAGE) analysis on 12%–18% acrylamide gradient gels with 8 M urea. Biological replicates were performed for independent cultures grown on separate days.

### Cloning of HA-tagged *CGL86* for expression in Chlamydomonas

CGL86-HA was cloned with the MoClo strategy as published (Crozet et al., 2018). The full-length CDS of *CGL86* (Cre12.g534450) lacking the endogenous stop codon was synthesized (IDT DNA) containing the first intron of *RBCS2* 5′-GTGAGTCGACGAGCAAGCCCGGCGGATCAGGCAGCGTGCTTGCAGATTTGACTTGCAACGCCCGCATTGTGTCGACGAAGGCTTTTGGCTCCTCTGTCGCTGTCTCAAGCAGCATCTAACCCTGCGTCGCCGTTTCCATTTGCAG-3′ between exons 3 and 4 of the original *CGL86* gene and cloned into pAGM1287 to obtain the compatible flanking restriction sites for MoClo cloning, yielding construct #272 L0_CDS_CGL86. The promoter region 824 bp upstream of the initiation codon was amplified by PCR with primers 5′-TTGAAGACAAGGAGCTAGTTCACACAAGGCGCCAGGC-3′ and 5′-TTGAAGACGCCATTGGTGGTGCACCTGCACGCAAG-3′, digested with BbsI and cloned into pICH41295, yielding construct #273 L0_prom_CGL86. For level 1, both plasmids were combined with plasmids pCM0-100 (3xHA), pCM0-119 (*RPL23* 3′UTR) from the Chlamydomonas MoClo kit (Crozet et al., 2018) and the destination vector pICH47742 (Weber et al., 2011). Restriction digest with BsaI and parallel ligation yielded level 1 constructs #274 L1_pCGL86_CGL86_HA_tL23. For level 2, construct #274 was combined with pCM1-01 (level 1 construct with the *aadA* gene conferring resistance to spectinomycin flanked by the *PSAD* promoter and terminator) from the Chlamydomonas MoClo kit, with plasmid pICH41744 containing the proper end-linker, and with destination vector pAGM4673 (Weber et al., 2011), digested with BbsI, and ligated to yield level 2 construct #275 L2_CGL86_HA_aadA (*aadA* + *CGL86* gene). Correct cloning was confirmed by Sanger sequencing.

### Miscellaneous

IPs were performed as described before (Willmund and Schroda, 2005; Rohr et al., 2019). The antibody against APE1 was raised by immunizing rabbits with mature APE1, lacking the N-terminal 42 amino acids corresponding to the cTP. For expression of tagged *CGL86*, the gene was synthesized lacking the endogenous introns but containing the first intron of *RBCS2* between the third and fourth exon. The gene lacking the stop codon was cloned downstream of the *CGL86* promoter and 5′ UTR sequences (824 bp) and upstream and in-frame with the sequence for a triple-HA tag via the MoClo strategy (Crozet et al., 2018). The construct was expressed in the Chlamydomonas uvm4 strain (Neupert et al., 2009). Aggregate preparation and polysome analyses were described before (Ries et al., 2017; Westrich et al., 2021). Protein extraction, SDS–PAGE, semi-dry blotting and immunodetection were carried out as described previously (Willmund and Schroda, 2005). Immunoblots were captured with the FUSION-FX7 Advance imaging system (PEQLAB) or the Intas ChemoStar version 6.0. All used antibodies are listed in Supplemental Table S1.

## Accession numbers

Genes mentioned in this study can be found under the following accession numbers at Phytozome: *RPL1* (uL1c, Cre02.g088900), *RPS11* (uS11c, Cre12.g514500), *EFTufA* (CreCp.g000700), *HSP22E* (Cre14.g617450), *HSP22F* (Cre14.g617400), *RCA1* (Cre04.g229300), *cpSRP54* (Cre11.g479750), *PsbB* (CreCp.g005100), *PsbC* (CreCp.g009700), *PsbH* (CreCp.g004800), *PsbZ* (CreCp.g004400), *PsaA* (CreCp.g004650), *PsaB* (CreCp.g006900), *RbcL* (CreCp.g007100), *AtpA* (CreCp.g007200), *AtpB* (CreCp.g008900), *PsbD* (CreCp.g009500), *ChlB* (CreCp.g000400), *ChlN* (CreCp.g008000), *APE1* (Cre16.g665250), and *CGL86* (Cre12.g534450).

For the raw data associated with Figures 2–9, all gene accession codes are in accordance with the GenBank/EMBL data libraries and given in the Supplemental Data Sets. The MS data are accessible via the ProteomeXchange Consortium PRIDE (Perez-Riverol et al., 2019) with the identifier PXD024949.

## Supplemental data 

The following materials are available in the online version of this article.

**Supplemental Data Set S1.** Data from array-based ribosome profiling experiments and control hybridizations with total RNA; Chlamydomonas data.

**Supplemental Data Set S2.** Data from array-based ribosome profiling experiments and control hybridizations with total RNA; tobacco data.

**Supplemental Data Set S3.** Changes of the chloroplast ribosome interactome during heat acclimation.

**Supplemental Data Set S4.** Summary of statistical analyses.

**Supplemental Figure S1.** Distribution of chloroplast ribosomes during heat acclimation.

**Supplemental Figure S2.** Reproducibility of translation output and RNA abundance data of the Chlamydomonas heat acclimation kinetics.

**Supplemental Figure S3.** Reproducibility of translation output between biological replicates of the heat kinetics and corresponding controls.

**Supplemental Figure S4.** Changes of relative footprint abundance between heat-treated and control samples in Chlamydomonas.

**Supplemental Figure S5.** Changes in relative RNA abundance between heat-treated and control samples in Chlamydomonas.

**Supplemental Figure S6.** Changes of relative TE between heat-treated and control samples in Chlamydomonas.

**Supplemental Figure S7.** Heat-induced ribosome redistribution on transcripts that encode PSII antenna subunits, all replicates.

**Supplemental Figure S8.** Heat causes local changes of chloroplast ribosome occupancy in Chlamydomonas.

**Supplemental Figure S9.** Additional plots for ribosome LRs.

**Supplemental Figure S10.** Reproducibility of ribosome footprint and RNA abundance data between replicates of *N. tabacum* heat treatment.

**Supplemental Figure S11.** Changes of relative translation output, RNA accumulation, and TE between heat-treated and control *N. tabacum* samples.

**Supplemental Figure S12.** Heat causes local changes of chloroplast ribosome occupancy in *N. tabacum*.

**Supplemental Figure S13.** Comparison of altered translation output during heat treatment between Chlamydomonas and *N. tabacum*.

**Supplemental Figure S14.** Reproducibility of chloroplast ribosome AP-MS.

**Supplemental Figure S15.** Control analyses for chloroplast ribosome affinity MS.

**Supplemental Figure S16.** Further controls for ribosome interactor validation.

**Supplemental Table S1.** Antibodies used in this study.

## Supplementary Material

koab317_Supplementary_Data

## Notes

R.T., F.R., and L.D.W. designed and performed most experiments and wrote parts of the text. Y.G. performed tobacco ribosome profiling experiments, C.H., J.H., and J.H.-R. conducted validation experiments of ribosome interactors. D.S. designed and performed microscopy. M.M. and Y.C. performed pulse labeling experiments. M.R. performed mass spectrometry measurements, helped with statistical analyses. R.Z. designed tobacco ribosome profiling experiments and wrote part of the manuscript. F.W. designed the project, evaluated data, and wrote the manuscript.

The author responsible for distribution of materials integral to the findings presented in this article in accordance with the policy described in the Instructions for Authors (https://academic.oup.com/plcell) is Felix Willmund (willmund@bio.uni-kl.de).
