# Supplementary material for: Fast and global reorganization of the chloroplast protein biogenesis network during heat acclimation
Source: Plant Cell. 2021 Dec 27;34(3):1075–99. doi: 10.1093/plcell/koab317 (PMC8894945; doi:10.1093/plcell/koab317)
Supplement: koab317_Supplementary_Data [file koab317_supplementary_data.zip › TPC2021RA00632R1 Supplemental Figures and Table FW.pdf]

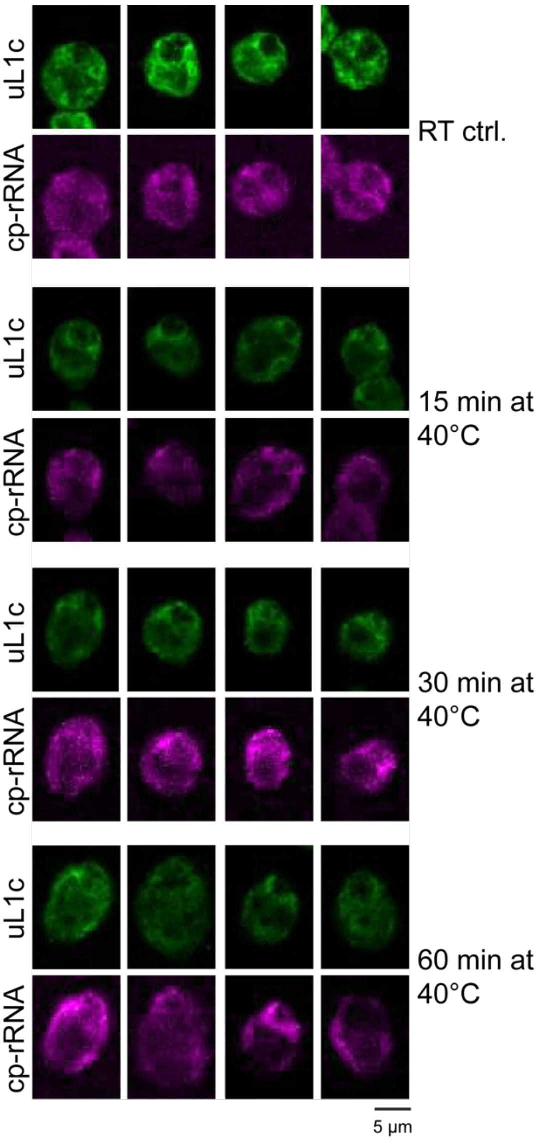

Supplemental Figure S1. Distribution of chloroplast ribosomes during heat acclimation.
Additional representative confocal microscopy images of Chlamydomonas cells during heat exposure to 40°C. Chloroplast ribosomes were visualized by staining with antibodies against uL1c (FITC, green) and a FISH probe against 70S rRNA (red). The contrast of the red channel was slightly but equally adjusted in all panels.
Supports Figure 1A.

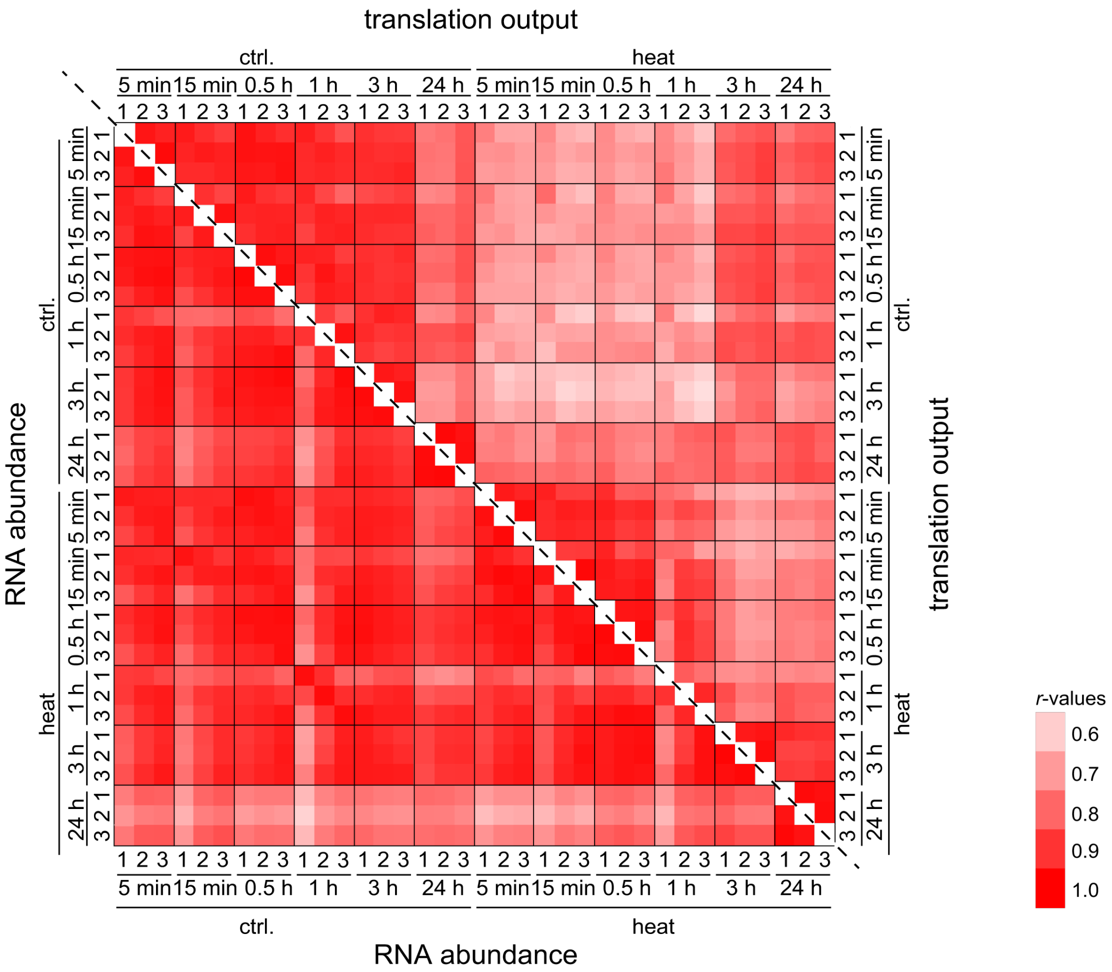

Supplemental Figure S2. Reproducibility of translation output and RNA abundance data of the Chlamydomonas heat acclimation kinetics

Heatmap representing Pearson's correlation coefficient values between the three independently processed biological replicates for all samples of the heat kinetics and controls, which were calculated based on normalized signal intensity of probes covering chloroplast reading frames (RF) of ribosome footprint (translation output; above diagonal line) or transcript (RNA; below diagonal line) samples. For individual Pearson's correlation coefficient and ANOVA's *p*-value (stating the significance of the regression) see Supplementary Data Set S1.

Supports Figure 2B.

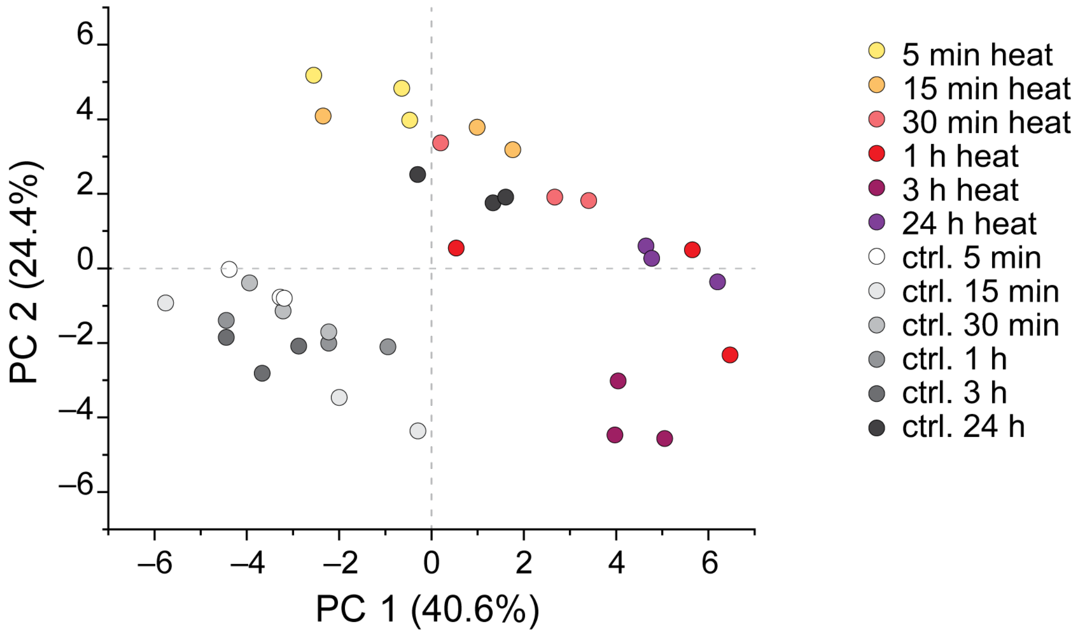

Supplemental Figure S3. Comparability of translation output between biological replicates of the heat kinetic and corresponding controls.
Principal component analysis of relative footprint abundance between heat-treated and control samples. Circles of the same color represent individual biological replicates. Note that samples after 24 h of heat or control treatment may vary due to some nutrient limitation, since the medium was not refreshed during the heat kinetics.
Supports Figure 2C.

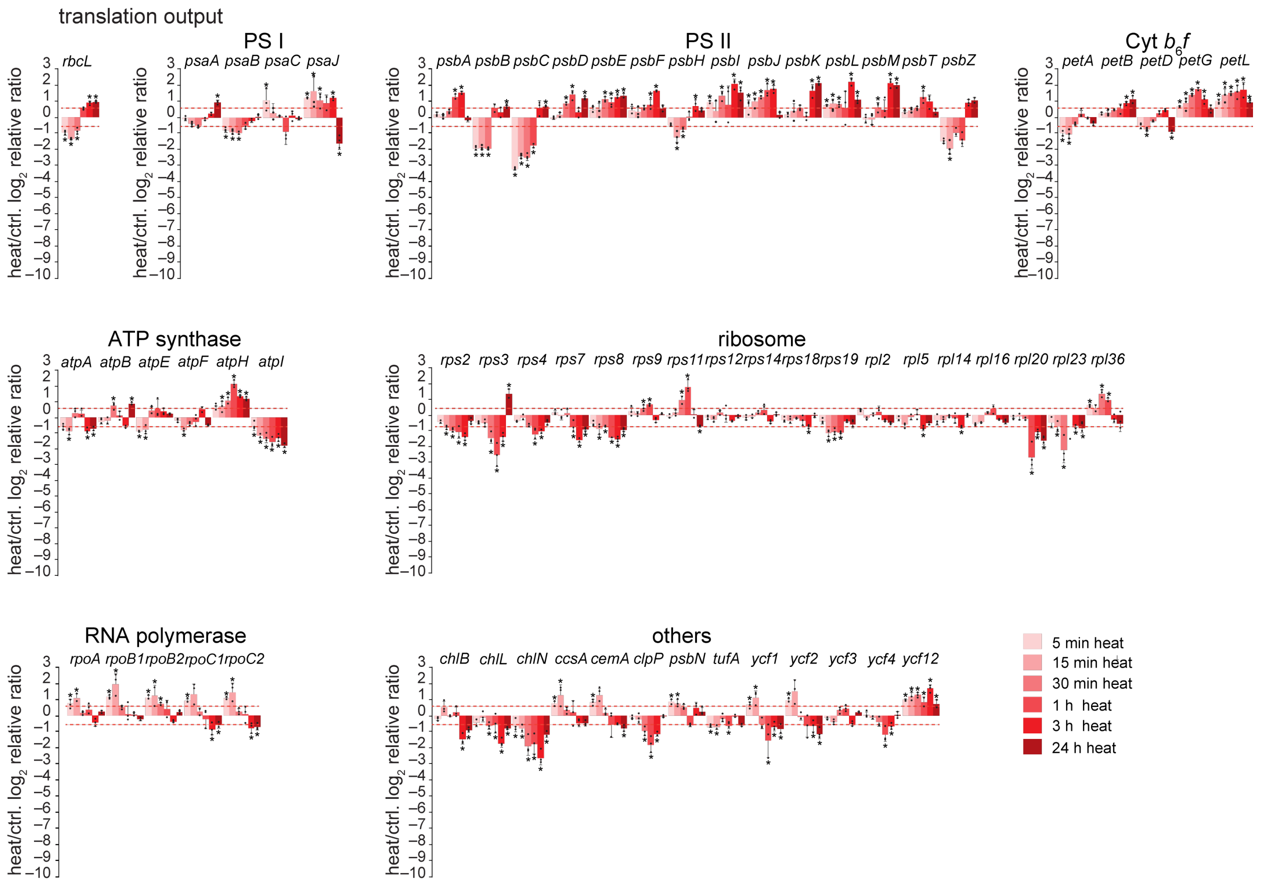

Supplemental Figure S4: Changes of relative footprint abundance between heat-treated and control samples in Chlamydomonas.
Comparison of the relative translation output of heat-exposed and control Chlamydomonas cells. Average ribosome footprint abundances were calculated for each known plastid RF and normalized to overall signal intensities per sample (giving "relative translation output"; for details see Methods). Bars represent the mean ratios (heat over control per time point of the kinetics) on a Log_2 scale for each chloroplast gene. Positive values indicate higher, negative values lower relative translation output during heat treatment compared to the room temperature control, respectively. Mean values are shown ± standard deviation from three independently grown biological replicates (note that for better visualization, error bars are only shown in one direction). Individual data points are shown as black circles. Asterisks indicate significant changes, based on a Welch's *t*-test and corrected for multiple testing according to Storey's *q*-value, for transcripts whose translation levels changed more than 1.5-fold between the two conditions (threshold indicated by a horizontal orange line). Genes are categorized in PS I, photosystem I; PS II, photosystem II; Cyt b_6f, cytochrome b_6f complex; ATP synthase; ribosome; RNA polymerase and others, all remaining chloroplast protein-coding genes.
Supports Figure 3B.

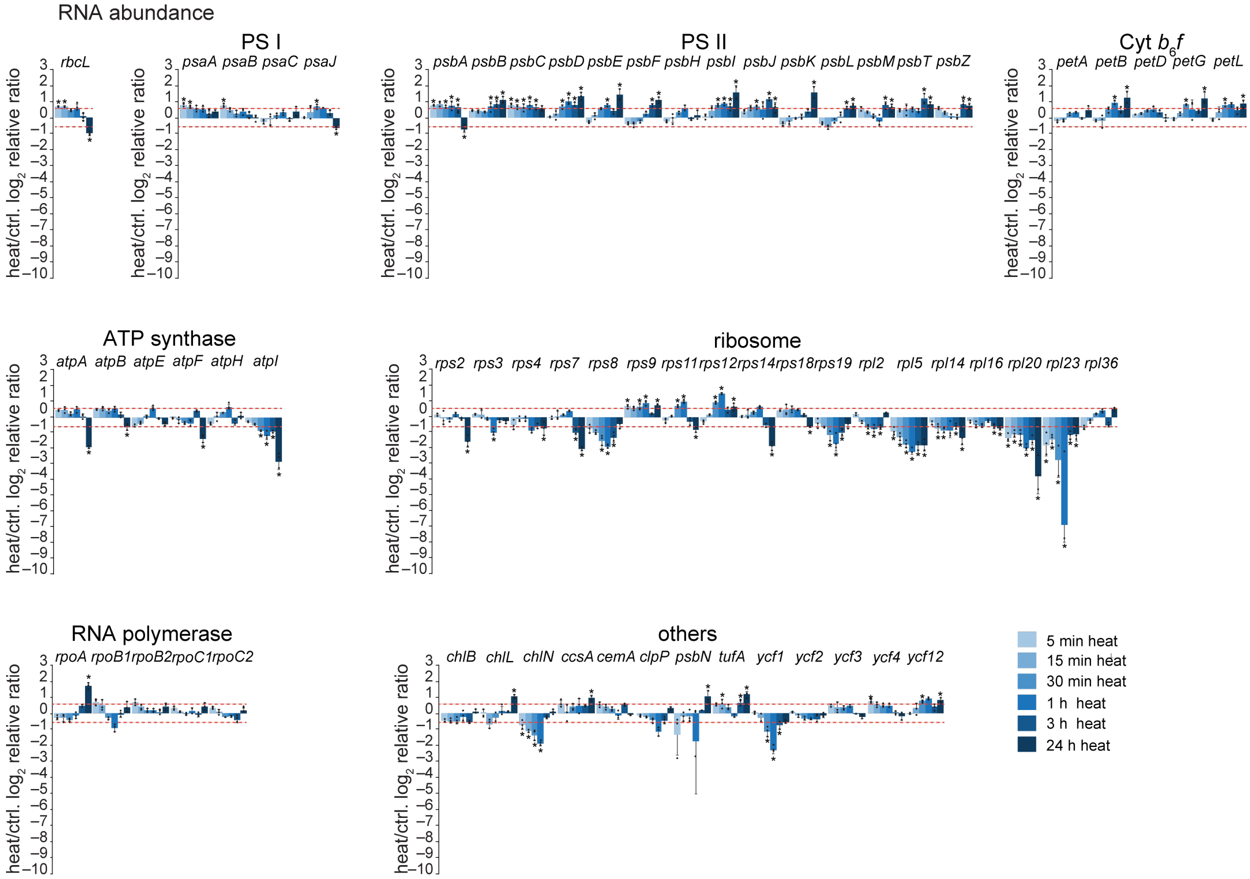

Supplemental Figure S5. Changes of relative RNA abundance between heat-treated and control samples in Chlamydomonas.

Comparison of the relative RNA accumulation of heat-exposed and room temperature control Chlamydomonas cells, as shown for translation output in Supplementary Figure S4.

Supports Figure 3B.

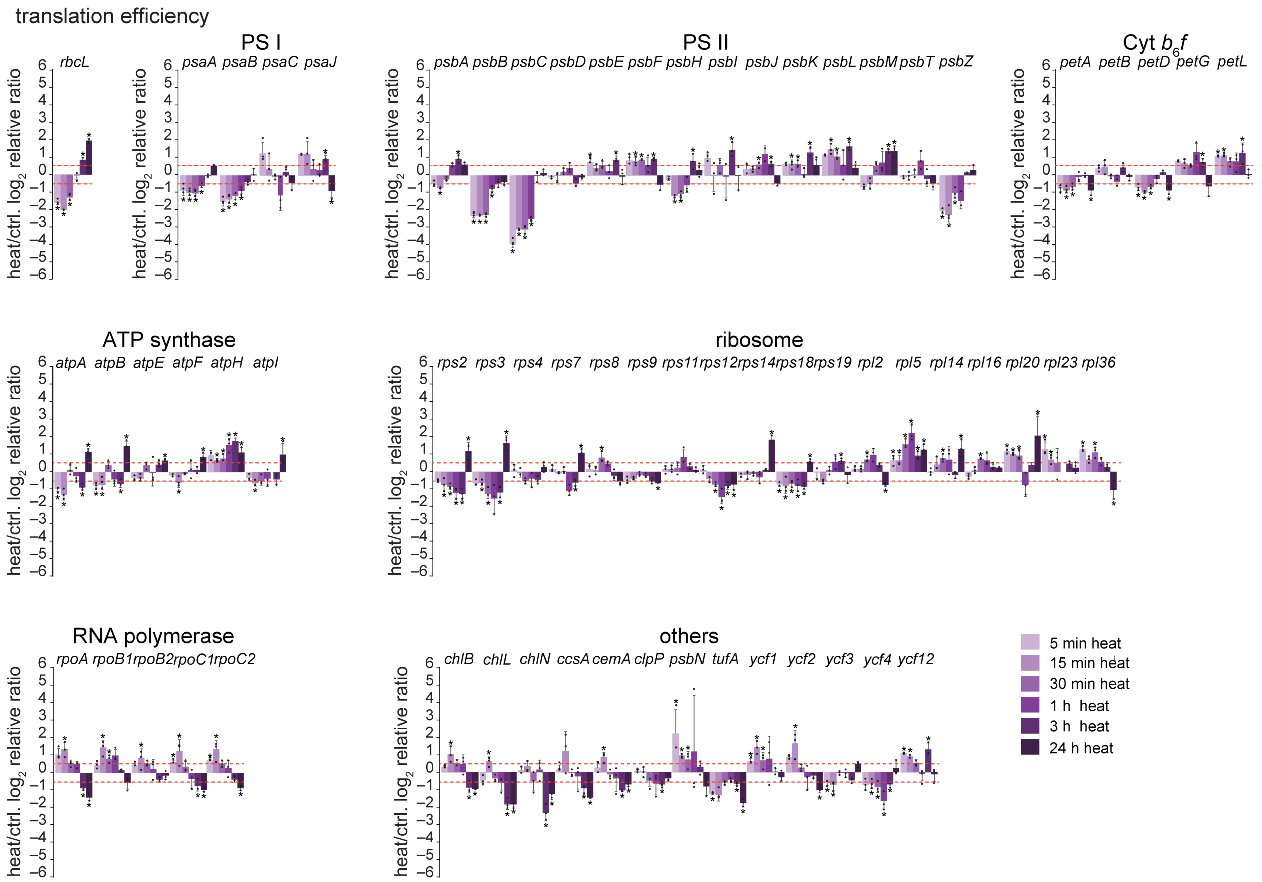

Supplemental Figure S6. Changes of relative translation efficiency between heat-treated and control samples in Chlamydomonas.

Comparison of the relative translation efficiency (TE) of heat exposed and room temperature control Chlamydomonas cells as shown for translation output in Supplementary Figure S4.

Supports Figure 3B.

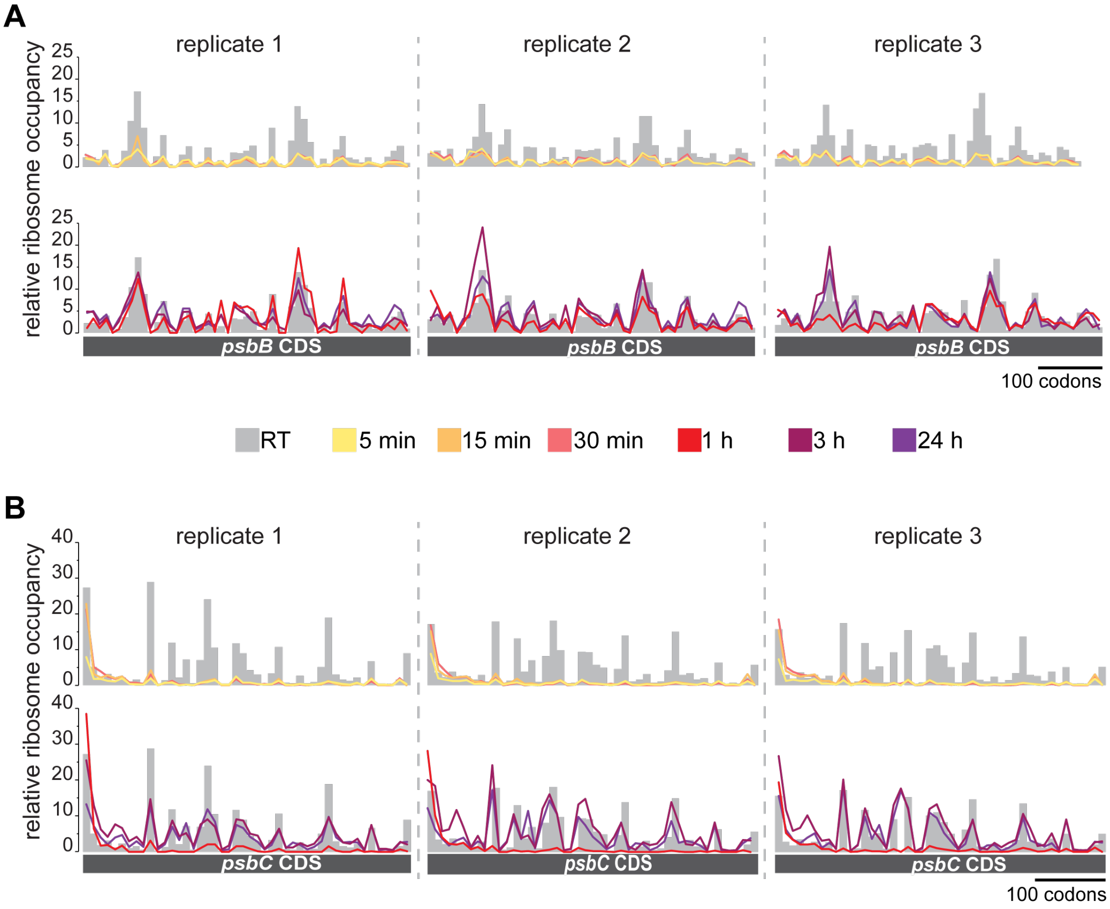

Supplemental Figure S7. Heat-induced ribosome redistribution on transcripts that encode PSII antenna subunits, all replicates

Side-by-side comparison of all three biological replicates (derived from independent cultures) to show the experimental variability of ribosome occupancy along the coding sequences (CDS) of (A) *psbB* and (B) *psbC*.

Supports Figure 4A.

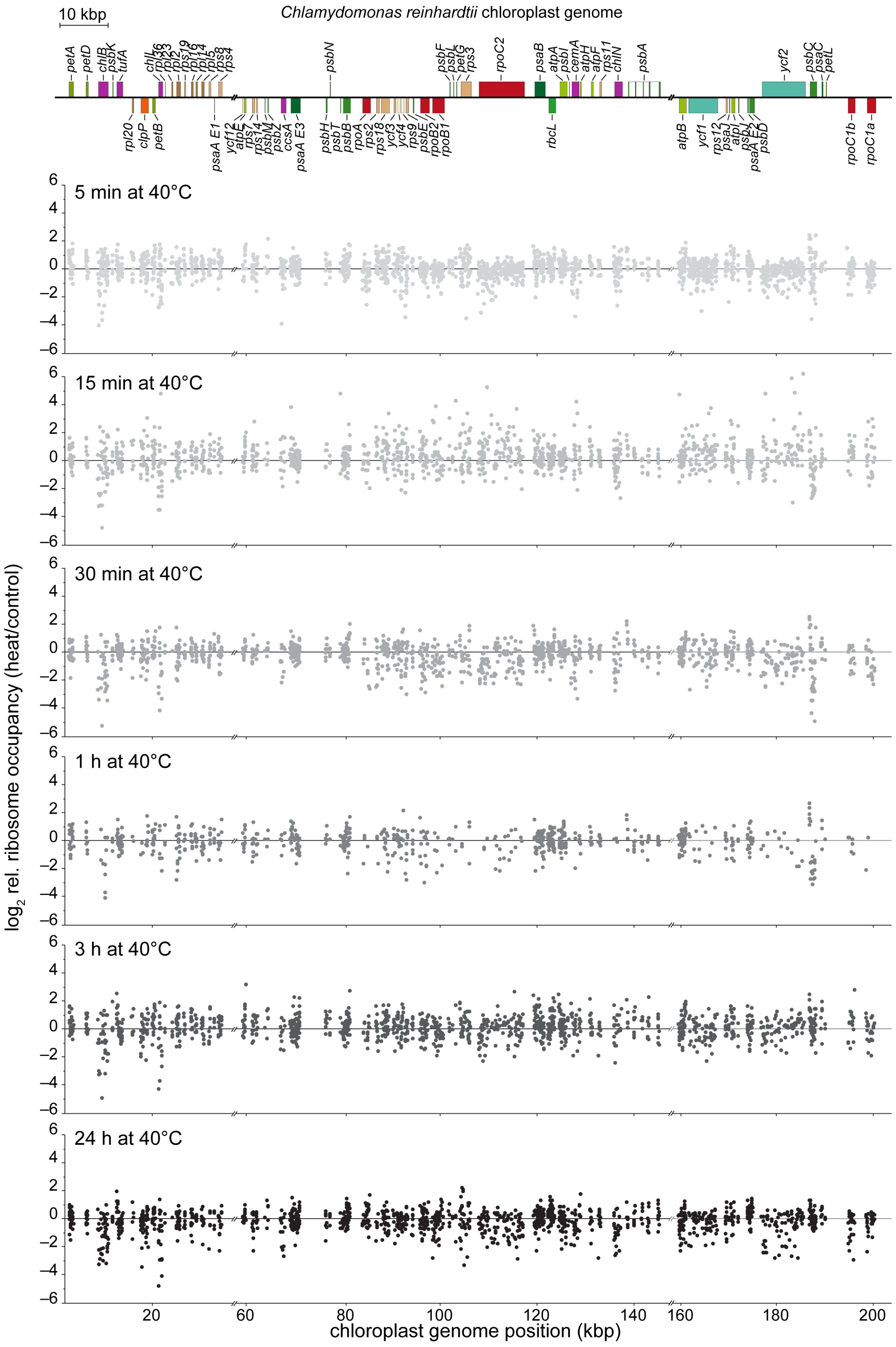

10 kbp
Chlamydomonas reinhardtii chloroplast genome
5 min at 40°C
15 min at 40°C
30 min at 40°C
1 h at 40°C
3 h at 40°C
24 h at 40°C
log2 rel. ribosome occupancy (heat/control)
chloroplast genome position (kbp)

Supplemental Figure S8. Heat causes local changes of chloroplast ribosome occupancy in Chlamydomonas.

Dot plot representing plastome-wide changes of relative ribosome occupancy within reading frames of heat-exposed cells in comparison to control. Top panel: Plastome map of known and characterized RFs. Map was drawn with OGDRAW (Lohse et al., 2013). For better visualization, spaces of large intergenic regions and the inverted repeat of the plastid genome were removed. Relative ribosome occupancies were calculated as described before (Schuster et al., 2019) and ratios were determined for heat-treated and control samples. Probes with very low signal intensities (<100) were not considered in this analysis. Plotted ratios between the two treatments are the means of three biological replicates and were plotted on a Log_2 scale.

Supports Figure 5.

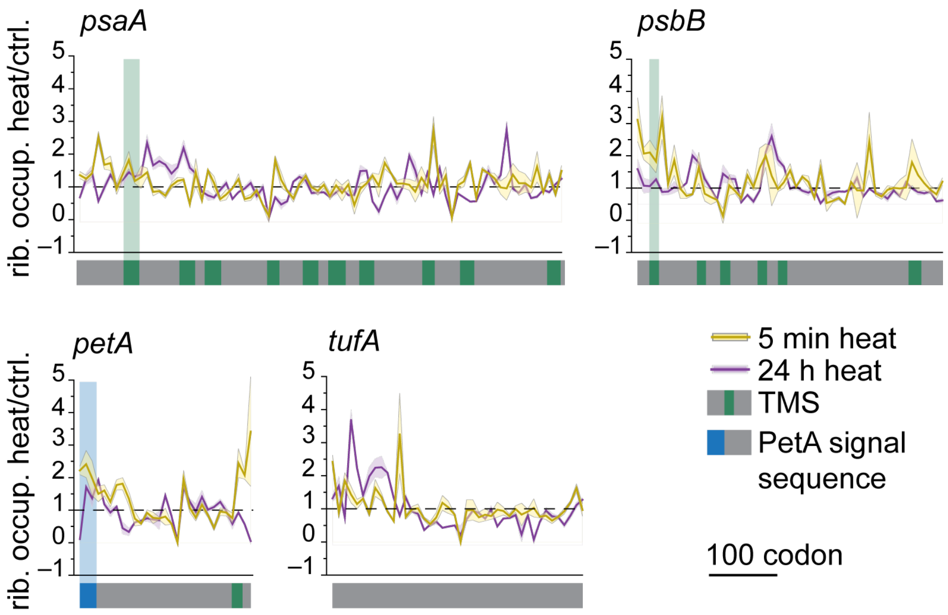

Supplemental Figure S9. Additional plots for ribosome loading ratios.
Additional ratio plots over selected RFs. Labeling as in Figure 5E.
Supports Figure 5E.

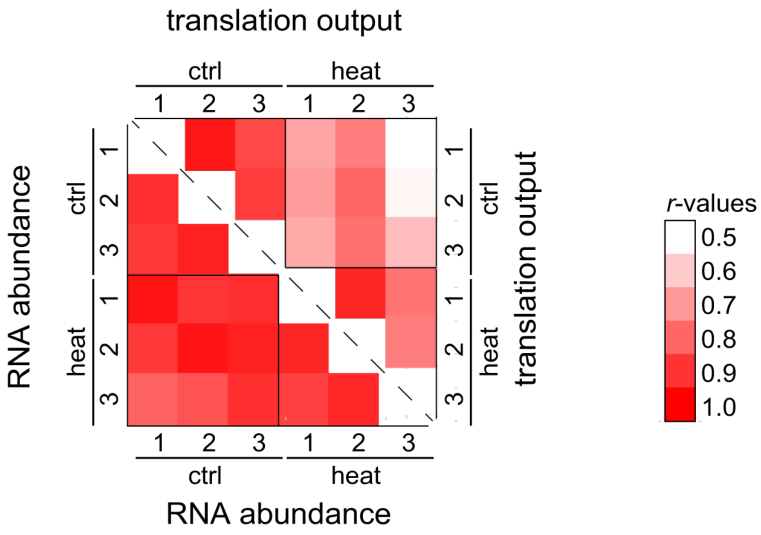

Supplemental Figure S10. Reproducibility of ribosome footprint and RNA abundance data between replicates of *Nicotiana tabacum* heat treatment.
Tobacco seedlings were exposed to 50°C for 90 min and an illumination of 350 µmol photons per m^{-2} s^{-1}. Heatmap represents Pearson's correlation coefficients between the three individually grown biological replicates. All samples of the heat kinetics and the controls were calculated based on the normalized signal intensity of all probes covering chloroplast RFs of transcript (RNA; below diagonal line) or ribosome footprint (translation output; above diagonal line) samples. For individual Pearson's correlation coefficients and ANOVA's *p*-value (stating the significance of the regression), see Supplementary Dataset 2.
Supports Figure 7.

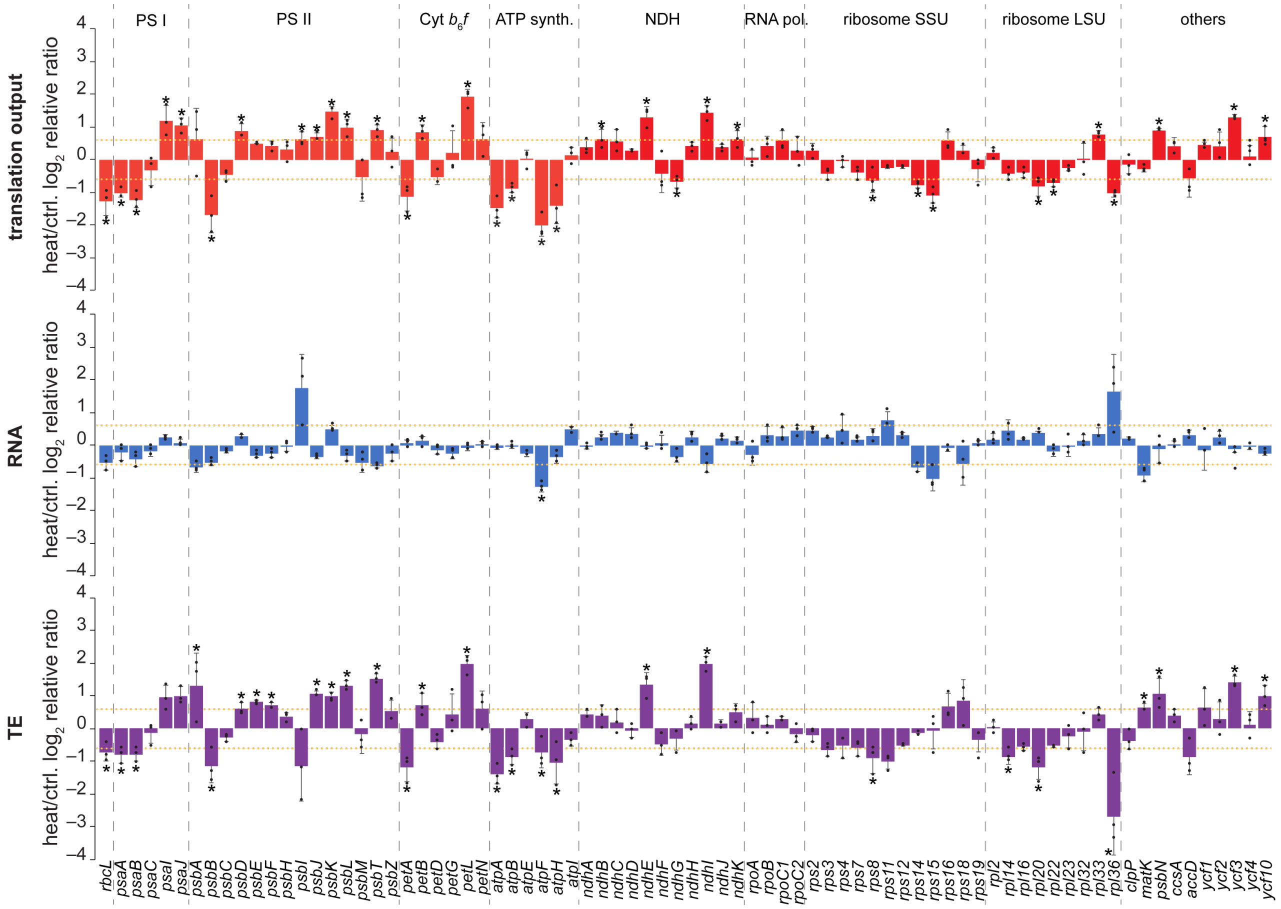

Supplemental Figure S11. Changes of relative translation output, RNA accumulation and TE between heat-treated and control *Nicotiana tabacum* samples

Comparison of the relative translation output, RNA and TE in heat-treated and room temperature control experiments with tobacco plants. Calculations for relative translation output, RNA abundance and TE were performed as described in Supplemental Figures S4-6. Data are shown as means ± standard deviation from three biologically independent samples (note that for better visualization, error bars are only shown in one direction); individual data points are shown as black circles. Asterisks indicate significant changes, based on a Welch's *t*-test and corrected for multiple testing according to Storey's *q*-value, for reading frames whose protein synthesis level changed more than 1.5-fold between two temperature conditions (threshold indicated by a horizontal orange line). Genes are categorized in PS I, photosystem I; PS II, photosystem II; Cyt b_6f, cytochrome b_6f complex; ATP synth., ATP synthase; ribosome SSU, LSU, small subunit, large subunit, respectively; RNA pol., RNA polymerase, NDH, NADH dehydrogenase-like complex, and others, all remaining chloroplast protein-coding genes.

Supports Figure 7.

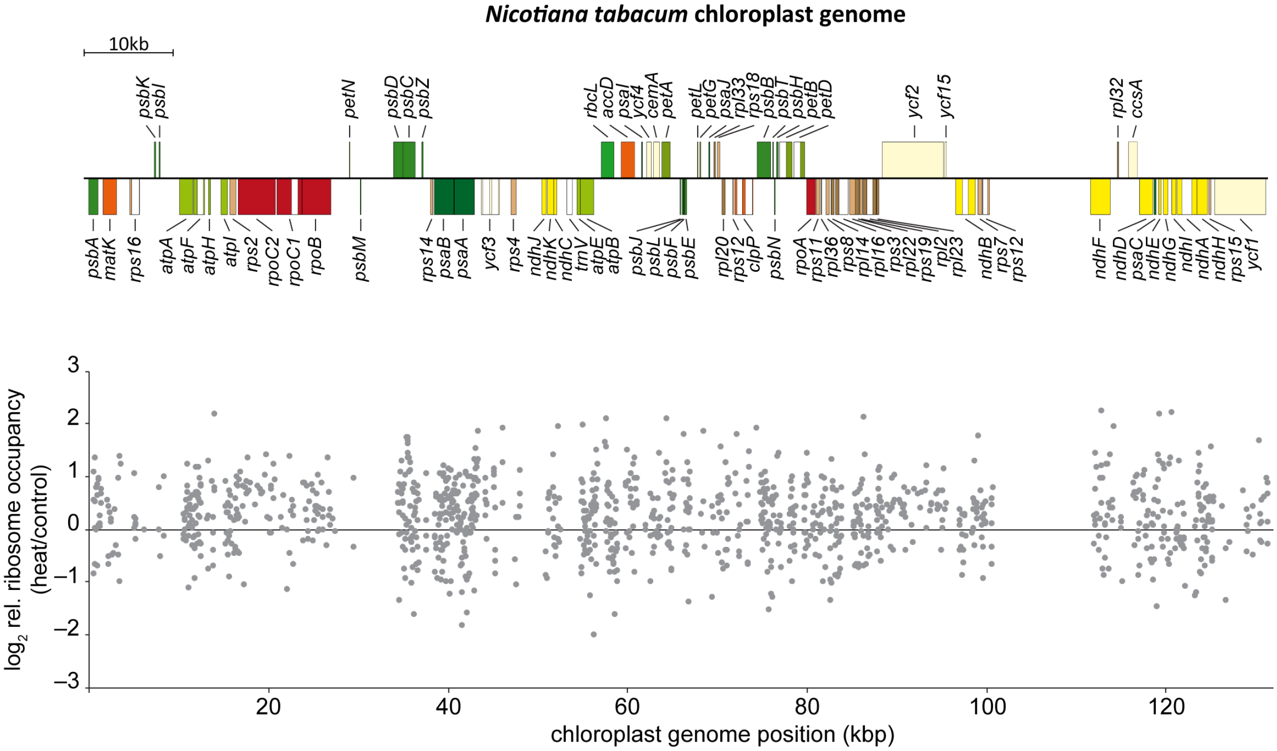

Supplemental Figure S12. Heat causes local changes of chloroplast ribosome occupancy in *Nicotiana tabacum*

Dot plots representing plastome-wide changes of relative ribosome occupancy within RFs of heat-exposed and control tobacco plants. Top panel: Plastome map, drawn with OGDRAW (Lohse et al., 2013). The inverted repeat was removed. Relative ribosome occupancies were calculated as described before (Schuster et al., 2019). The ribosome footprint signal intensity of each probe located within a protein coding RF was normalized to the sum of the signal intensities of all probes in the respective RF for heat treated and control samples, respectively. Ratios between the two treatments represent mean values of three biological replicates and are plotted on a Log_2 scale.

Supports Figure 7.

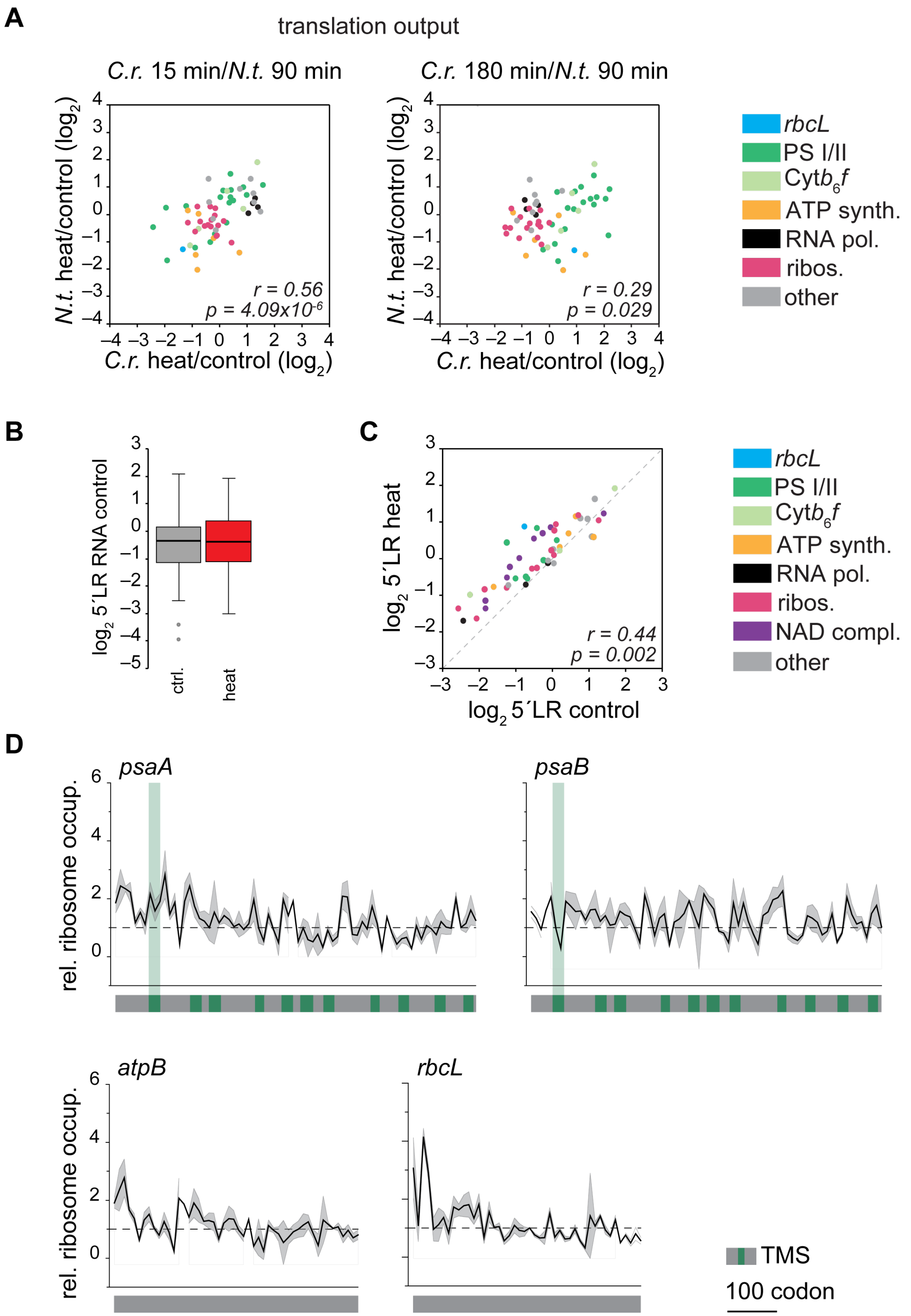
A
translation output
C.r. 15 min/N.t. 90 min
C.r. 180 min/N.t. 90 min
N.t. heat/control (log2)
C.r. heat/control (log2)
r = 0.56
p = 4.09x10-6
r = 0.29
p = 0.029
rbcL
PS I/II
Cytb6f
ATP synth.
RNA pol.
ribos.
other
B
log2 5´LR RNA control
ctrl.
heat
C
log2 5´LR heat
log2 5´LR control
r = 0.44
p = 0.002
NAD compl.
D
psaA
psaB
atpB
rbcL
rel. ribosome occup.
TMS
100 codon

Supplemental Figure S13. Comparability of altered translation output during heat treatment between Chlamydomonas and tobacco.
(A) Scatterplots for the interspecies comparison of altered translation output in heat-treated samples. Relative ribosome footprint abundance ratios between heat and control are plotted for Chlamydomonas *(C.r.)* on the x-axis versus tobacco (*N.t.*) ratios on the y-axis. Left panel, comparison of short-term 15 min heat exposure in Chlamydomonas and 90 min heat exposure in tobacco. Right panel, comparison of long-term 180 min heat exposure in Chlamydomonas and 90 min heat exposure in tobacco. All values represent the mean of three biologically independent samples. Pearson's correlation coefficients are given in the graph. (B) Probe intensities of the first ~60 codons for the mRNA samples show that the increased 5'LRs in tobacco during heat (Figure 7F) are not the consequence of fragmented mRNAs. (C) 5'LRs within individual transcripts plotted between heat-treated tobacco samples and room temperature control. Functional groups of genes are color-coded as indicated in the legend to the right. All values represent the mean of three biologically independent samples. Gene groups are as in Figures S4 and S11. (D) Additional ratio plots over selected RFs of relative ribosome occupancy between heat treated and control samples reveal local changes of elongation in tobacco. The relative ribosome footprint occupancy was calculated as the fraction of probe signal to the summed signal for each RF and condition. The dashed line indicates the level with no change between heat and control samples. Error bars are plotted as ribbon depicting standard deviations calculated from three independent biological replicates. Transmembrane domain segments (TMS) are marked in green, with the first TMS extended.
Supports Figure 7.

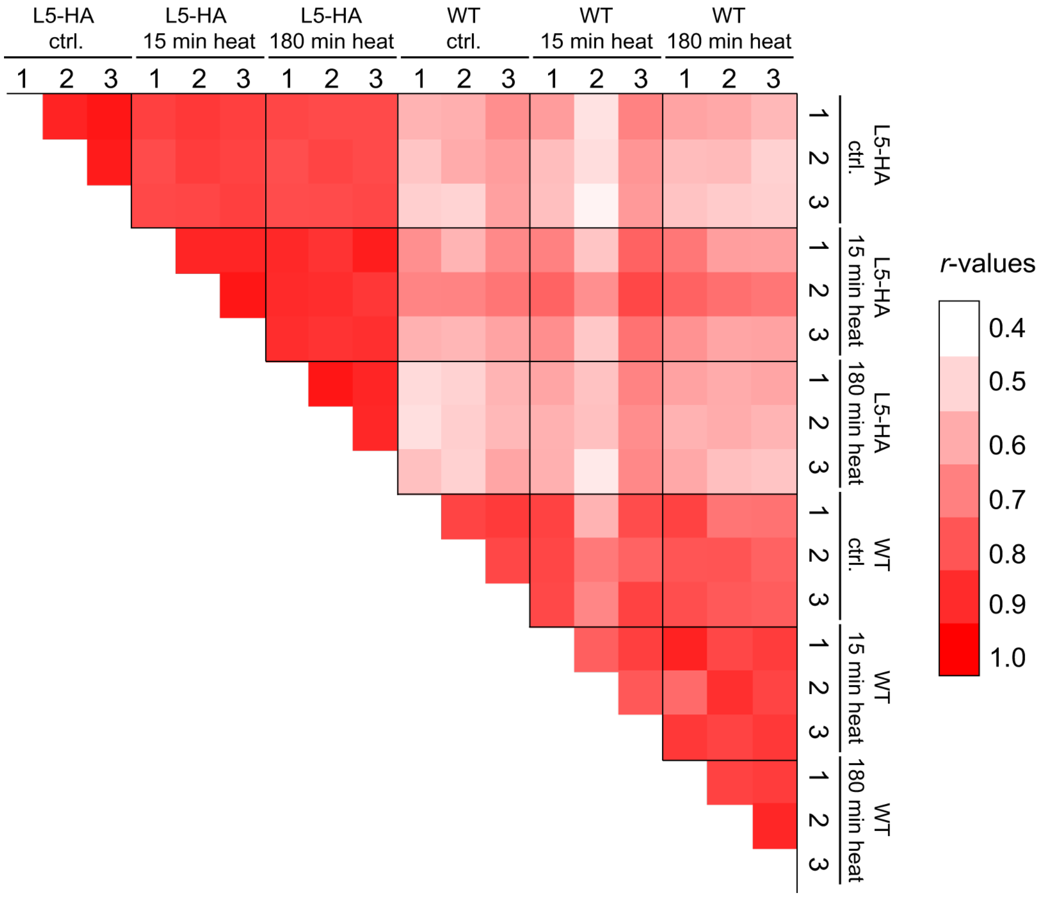

Supplemental Figure S14. Reproducibility of chloroplast ribosome AP-MS.
Pearson correlation coefficients between all affinity purification-mass spectrometry experiments with tagged Rpl5-HA (L5-HA) and the untagged controls (WT) from room temperature (ctrl.) and heat-treated for 15 min and 180 min. Column correlation is based on filtered Log_2-transformed LFQ values set (see Supplemental Data Set S3).
Supports Figure 8.

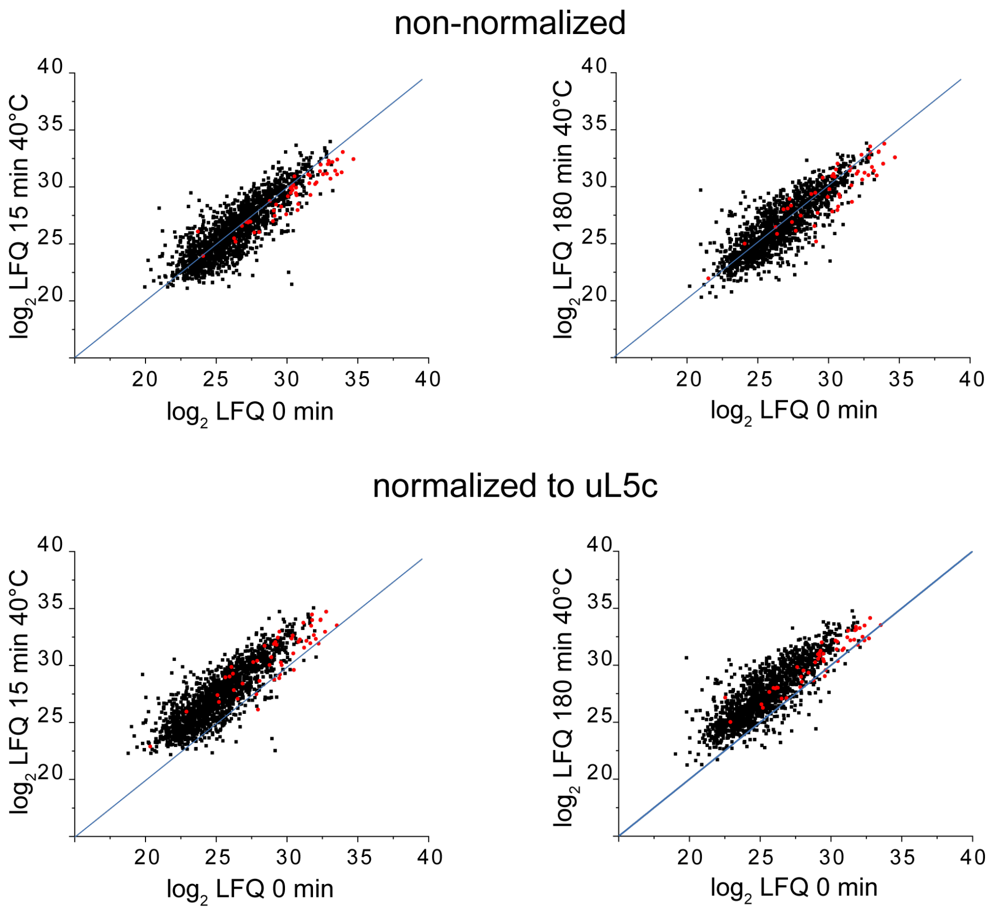

Supplemental Figure S15. Control analyses for chloroplast ribosome affinity mass spectrometry. Scatterplot of LFQ values from all quantified proteins, comparing the control time point with heat treatment for 15 and 180 min. Red dots are the chloroplast ribosomal proteins. In the lower panel, the values are corrected for the deviation of bait abundance, which shifts the whole dataset away from the middle line. Supports Figure 8.

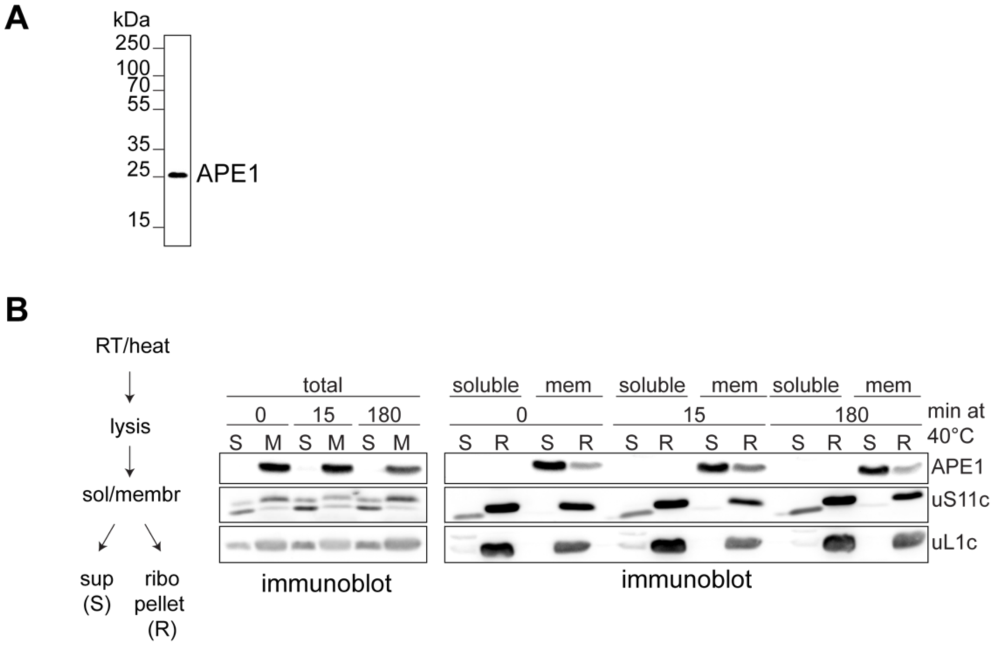

Supplemental Figure S16. Further controls of ribosome interactor validation.
(A) Specific antiserum detecting APE1 in Chlamydomonas cells was generated by immunizing rabbits against recombinant mature Chlamydomonas APE1. The specificity of the antibody was tested by immunoblotting whole cell Chlamydomonas extracts with the APE1 antiserum. (B) Ribosome co-sedimentation of APE1 in Chlamydomonas cells under ambient temperatures (0 min) and 15 and 180 min of heat exposure at 40°C. Cell lysates were separated into soluble and membrane (mem) fractions by centrifugation at 16,000 *g* for 15 min. Total samples of these fractions are shown in the left panel. Ribosomal complexes (R) were separated from non-ribosomal fractions (S) by centrifugation through a 25% (w/v) sucrose cushion, fractions were separated on a 12% SDS-polyacrylamide gel and analyzed by immunoblotting.
Supports Figure 10.

Supplemental Table 1. Antibodies used in this study

Target	Source	Reference
uL1c/PRPL1	own production	(Ries et al., 2017)
HA	monoclonal mouse antibody, Sigma Aldrich	-
cpSRP54	own production	(Rohr et al., 2019)
uS11c/Rps11	own production	(Westrich et al., 2021)
uL37/RPL37	J.-D.Rochaix/S. Ramundo	(Ramundo et al., 2014)
HSP22E/F	M. Schroda	(Rütgers et al., 2017)
TufA	J.-D.Rochaix/S. Ramundo	(Ramundo et al., 2014)
RCA1	polyclonal rabbit antibody, Agrisera (#AS10 700)	-
HSP70B	M. Schroda	(Schroda et al., 1999)
PsbA/D1	polyclonal rabbit antibody, Agrisera (#AS05 084)	-
PsbD/D2	polyclonal rabbit antibody, Agrisera (#AS06 146)	-
PbsB/CP47	polyclonal rabbit antibody, Agrisera (#AS04 038)	-
PbsC/CP43	polyclonal rabbit antibody, Agrisera (#AS11 1787)	-
AtpB	own production, polyclonal rabbit antibody, raised against the full-length chloroplast AtpB protein from Chlamydomonas	this study
cpHsp70	polyclonal rabbit antibody against Arabidopsis chloroplast Hsp70, Agrisera ((#AS08 348)	(Lemaire and Wollman, 1989)
APE1	own production, raised against the mature chloroplast APE1 protein (lacking the 42 amino acids of the chloroplast transit peptide) from Chlamydomonas	this study
CGE1	M. Schroda	(Schroda et al., 2001)
Cytochrome *f*		(Pierre and Popot, 1993)

References

Crozet, P., Navarro, F.J., Willmund, F., Mehrshahi, P., Bakowski, K., Lauersen, K.J., Perez-Perez, M.E., Auroy, P., Gorchs Rovira, A., Sauret-Gueto, S., Niemeyer, J., Spaniol, B., Theis, J., Trösch, R., Westrich, L.D., Vavitsas, K., Baier, T., Hübner, W., de Carpentier, F., Cassarini, M., Danon, A., Henri, J., Marchand, C.H., de Mia, M., Sarkissian, K., Baulcombe, D.C., Peltier, G., Crespo, J.L., Kruse, O., Jensen, P.E., Schroda, M., Smith, A.G., and Lemaire, S.D. (2018). Birth of a Photosynthetic Chassis: A MoClo Toolkit Enabling Synthetic Biology in the Microalga *Chlamydomonas reinhardtii*. ACS Synth Biol **7,** 2074-2086.

Lemaire, C., and Wollman, F.A. (1989). The chloroplast ATP synthase in Chlamydomonas reinhardtii. II. Biochemical studies on its biogenesis using mutants defective in photophosphorylation. J Biol Chem **264,** 10235-10242.

Lohse, M., Drechsel, O., Kahlau, S., and Bock, R. (2013). OrganellarGenomeDRAW-a suite of tools for generating physical maps of plastid and mitochondrial genomes and visualizing expression data sets. Nucleic Acids Res **41,** W575-581.

Pierre, Y., and Popot, J.L. (1993). Identification of two 4-kDa miniproteins in the cytochrome b6f complex from *Chlamydomonas reinhardtii*. C R Acad Sci III **316,** 1404-1409.

Ramundo, S., Casero, D., Muhlhaus, T., Hemme, D., Sommer, F., Crevecoeur, M., Rahire, M., Schroda, M., Rusch, J., Goodenough, U., Pellegrini, M., PerezPerez, M.E., Crespo, J.L., Schaad, O., Civic, N., and Rochaix, J.D. (2014). Conditional Depletion of the Chlamydomonas Chloroplast ClpP Protease Activates Nuclear Genes Involved in Autophagy and Plastid Protein Quality Control. Plant Cell **26,** 2201-2222.

Ries, F., Carius, Y., Rohr, M., Gries, K., Keller, S., Lancaster, C.R.D., and Willmund, F. (2017). Structural and molecular comparison of bacterial and eukaryotic trigger factors. Sci Rep **7,** 10680.

Rohr, M., Ries, F., Herkt, C., Gotsmann, V.L., Westrich, L.D., Gries, K., Trösch, R., Christmann, J., Chaux, F., Jung, M., Zimmer, D., Mühlhaus, T., Sommer, F.K., Schroda, M., Keller, S., Möhlmann, T., and Willmund, F. (2019). The role of plastidic trigger factor serving protein biogenesis in green algae and land plants. Plant Physiol.

Rütgers, M., Muranaka, L.S., Mühlhaus, T., Sommer, F., Thoms, S., Schurig, J., Willmund, F., Schulz-Raffelt, M., and Schroda, M. (2017). Substrates of the chloroplast small heat shock proteins 22E/F point to thermolability as a regulative switch for heat acclimation in *Chlamydomonas reinhardtii*. Plant Mol Biol **95,** 579591.

Schroda, M., Vallon, O., Wollman, F.A., and Beck, C.F. (1999). A chloroplast-targeted heat shock protein 70 (HSP70) contributes to the photoprotection and repair of photosystem II during and after photoinhibition. Plant Cell **11,** 1165-1178.

Schroda, M., Vallon, O., Whitelegge, J.P., Beck, C.F., and Wollman, F.A. (2001). The chloroplastic GrpE homolog of Chlamydomonas: two isoforms generated by differential splicing. Plant Cell **13,** 2823-2839.

Schuster, M., Gao, Y., Schöttler, M.A., Bock, R., and Zoschke, R. (2019). Limited Responsiveness of Chloroplast Gene Expression during Acclimation to High Light in Tobacco. Plant Physiol.

Westrich, L.D., Gotsmann, V.L., Herkt, C., Ries, F., Kazek, T., Trosch, R., Armbruster, L., Mühlenbeck, J.S., Ramundo, S., Nickelsen, J., Finkemeier, I., Wirtz, M., Storchova, Z., Räschle, M., and Willmund, F. (2021). The versatile interactome of chloroplast ribosomes revealed by affinity purification mass spectrometry. Nucleic Acids Res **49,** 400-415.
